# SMARTCLOTH Prototype for Dietary Management in Patients With Diabetes Mellitus: Tutorial on Human-Centered Design Methodology for Health Care Hardware Development

**DOI:** 10.2196/75744

**Published:** 2026-01-21

**Authors:** Jose M Palomares, Rafael Molina-Luque, Fernando León-García, Irene Casares-Rodríguez, María García-Rodríguez, María Pilar Villena Esponera, Guillermo Molina-Recio

**Affiliations:** 1 Lifestyles, Innovation and Health Associated Group, Maimonides Institute for Biomedical Research (IMIBIC), Avd. Menéndez Pidal, N/N, Córdoba, 14004, Spain, 34 957218101; 2 Advanced Informatics Research Group (GIIA), University of Cordoba (UCO), Córdoba, Spain; 3 Department of Electronic and Computer Engineering, University of Cordoba (UCO), Córdoba, Spain; 4 Department of Nursing, Pharmacology, and Physiotherapy, University of Cordoba (UCO), Córdoba, Spain; 5 Faculty of Health Sciences, Atlantic-Mediterranean Technological University (UTAMED), Málaga, Spain; 6 Faculty of Arts and Social Sciences, International University of La Rioja (UNIR), Logroño, Spain

**Keywords:** type 1 diabetes mellitus, type 2 diabetes mellitus, dietary adherence, user-centered design: usability, digital health

## Abstract

**Background:**

Developing user-centered digital health hardware requires systematic design methods applicable across clinical contexts. As diabetes mellitus continues to rise globally and contributes to morbidity, mortality, and costs, effective nutritional management remains essential—yet adherence is often poor. Digital health interventions grounded in human-centered design may enhance adherence by better aligning solutions with patients’ real needs.

**Objective:**

This tutorial aims to provide replicable guidance on applying the design thinking approach to health care hardware development, illustrated through the design, development, and preliminary usability evaluation of SMARTCLOTH (GA-16: Lifestyles, Innovation, and Health), a smart tablecloth prototype intended to facilitate dietary management and support adherence to nutritional recommendations among individuals with diabetes.

**Methods:**

We demonstrate a systematic design thinking approach adaptable to other hardware contexts, using the Double Diamond model. In mapping, we performed a structured preassessment to define project scope and feasible functionalities. To characterize end user needs, we conducted 6 in-depth interviews with health care professionals and applied persona, empathy map, and customer journey map tools. In exploring, 5 focus groups (patients and diabetes educators) identified barriers, facilitators, and desired functionalities for dietary self-management. In building, we created low- and high-fidelity wireframes and interactive web prototypes using Phaser 3 (HTML5/JS) to simulate a kitchen workspace for meal assembly. In testing, 7 patients with different diabetes profiles participated in 3 iterative usability sessions. Using think-aloud, video analysis, and structured tasks, we documented completion times, errors, and the level of required assistance, enabling refinements. Development progressed through 15 internal versions and 3 user-tested prototypes with real-time adjustments when feasible.

**Results:**

Interviews and focus groups yielded three user profiles guiding design: (1) adolescents with type 1 diabetes navigating social and dietary challenges, (2) working-age adults with type 2 diabetes who were motivated but inconsistent, and (3) older adults with type 2 diabetes showing low adherence due to entrenched habits. Iterative usability testing indicated that the system was intuitive, with improvements in layout, labeling, and navigation. Quantitative metrics showed refinement, with simple tasks being completed in under 1 minute in later iterations, while complex meal simulations took longer. Error rates and required guidance decreased as prototypes evolved. Qualitative feedback highlighted clarity, motivational value, and educational potential, while older participants requested larger text and simplified controls. Despite usability gains, motivational barriers persisted among low-adherence older adults.

**Conclusions:**

This tutorial demonstrates that systematic human-centered design can yield feasible and well-accepted digital health hardware. SMARTCLOTH emerged as a promising tool for dietary management in diabetes, though effectiveness and clinical outcomes were not evaluated. The methodology can be adapted by teams developing hardware for chronic disease management.

## Introduction

Developing effective digital health hardware—such as wearable devices, smart medical equipment, or assistive technologies—presents unique methodological challenges that distinguish it from software-based interventions. Hardware development requires balancing user needs with physical constraints, manufacturing feasibility, and cost considerations, while ensuring usability across diverse populations. Human-centered design (HCD) and design thinking (DT) methodologies offer structured approaches to navigate these challenges, yet practical, step-by-step guidance on applying these methods to health care hardware development remains limited in the literature. This tutorial addresses this gap by providing detailed methodological guidance, illustrated through a real-world development case.

Diabetes mellitus (DM) is a chronic disease in which glucose metabolism is altered due to increased resistance to insulin action or low insulin production [1]. The prevalence of this pathology has increased significantly over the last few decades and will continue to do so in the coming decades. Data show that in 2024, an estimated 589 million adults were living with DM, a figure that could reach 853 million by 2050. More than 90% of these cases are type 2 diabetes mellitus (T2DM), making this disease a global public health challenge [2,3].

In addition to the significance of prevalence figures, it is worth noting that DM places a high demand on health care personnel due to the multiple comorbidities it can cause, including nephropathies, vasculopathies, and neuropathies, among others [4], with disease management playing a crucial role in this context. Diabetes management is a dynamic and long-term process that begins at diagnosis, when initial education and treatment are introduced, and continues with daily self-management tasks such as diet, medication adherence, and glucose monitoring. Periodic follow-up is also essential to prevent complications and adapt treatment as the disease progresses. In this context, diabetes self-management education and support should be provided at diagnosis, during critical transitions, and on an ongoing basis throughout the care process [5]. Diabetes self-management education and support contributes to both daily management and the acquisition of self-care skills, which are crucial for long-term adherence. Among lifestyle behaviors, nutrition is particularly influential [6,7], yet adherence to healthy dietary patterns remains inadequate in many patients [8,9]. Our research group focuses on the stage of learning and consolidating dietary self-management skills and on supporting sustained adherence through innovative tools adapted to patients’ contexts that improve their clinical situation.

Among dietary strategies, carbohydrate counting stands out as the cornerstone of nutritional management in type 1 diabetes mellitus (T1DM), as it enables the adjustment of insulin doses [10]. However, its complexity often hinders adherence. The rapid growth of digital technologies has created new opportunities in this field. Mobile health apps have been shown to support lifestyle changes and chronic disease management [11-13]. In diabetes, both generalist apps (eg, MyFitnessPal [Mike Lee] and CalorieMama [Azumio Inc]) and T1DM-specific apps (eg, CarbAndMove, SocialDiabetes [SocialDiabetes SL], and Carb Manager [Wombat Apps LLC]) are widely available. Nevertheless, they share essential limitations, including low usability and adherence [14,15], limited involvement of health care professionals in their development [16], technical problems, and poor integration with glucose-monitoring devices [17,18]. This fragmentation often forces patients to use multiple apps simultaneously, complicating management and increasing the risk of abandonment.

Recent reviews also highlight that evidence of their effectiveness remains inconsistent, and many tools fail to adequately meet patients’ real needs [19,20]. Successful solutions must therefore actively involve end users in the design process and incorporate features such as educational content, monitoring tools, goal setting, gamification, reminders, and social interaction options [21-23]. Positioning these initiatives within health behavior change frameworks [23-25] provides a comprehensive foundation for understanding how capability, opportunity, and motivation interact to enable sustained dietary change. In this regard, technological tools should not only support day-to-day management but also strengthen patients’ ability to learn, consolidate, and adhere to effective self-care strategies over time [26].

This complex clinical challenge—requiring sustained user engagement, multidisciplinary collaboration, and hardware-software integration—provides an ideal context for demonstrating the systematic application of HCD methodology. The following sections present a replicable approach that other teams can adapt to their own hardware development challenges in chronic disease management.

In this scenario, HCD emerges as an innovation method particularly suited to the development of solutions in the highly ambiguous field of health care. It focuses on creating high-value solutions through a people-centered approach that leverages diversity of thought, creativity, and ethnographic insights, which are refined through iterative testing [27,28]. The iterative nature of HCD reduces risks in terms of time and budget, mitigates cognitive biases among researchers, empowers individuals and teams, and enhances both creative confidence and solution quality [29-33]. Despite these advantages, HCD remains relatively new and is inconsistently applied in health care innovation [34]. This fact is particularly relevant for addressing health care disparities, including the digital divide, which can be mitigated through collaborative solutions developed in partnership with patients and stakeholders [35,36].

HCD also complements classical scientific approaches by improving the translation of evidence into practice and identifying opportunities for intervention in target populations [33,37]. Although it is often used interchangeably with DT [27,30,34,38], important distinctions exist between the two. While both are iterative and user-centered, HCD applies more rigorous methods aimed at usability and user satisfaction in digital health products. In contrast, DT has a broader scope that emphasizes innovation across diverse contexts [37-39]. In short, HCD can be considered a comprehensive framework, while DT operates as a flexible strategy for problem-solving within that framework [40].

At the same time, designing any logical or physical system is inherently complex, shaped by both enabling and constraining factors. The classical system design cycle outlines 5 main stages, namely objectives, research, requirements, design, and evaluation, yet traditionally involves users only in the final evaluation phase [41]. In practice, the process is iterative, with later phases informing adjustments to earlier ones. However, excluding users from early stages often results in products that lack adequate user experience, making people feel uncomfortable when using them with tools that were not developed according to their real needs and requirements.

For this reason, some methodologies have evolved to involve end users and stakeholders throughout the design cycle [29-31,34,38,39]. HCD has proven particularly effective for developing systems and interfaces tailored to users’ needs while aligning with institutional objectives [32,33,37,38]. Because users are deeply engaged throughout the process, the resulting interfaces provide a better fit and more satisfying usability experience [27,28]. User input can be collected through both direct methods (eg, questionnaires and interviews) and indirect approaches (eg, observation of prototype interactions and session recordings), complemented by quantitative measures, such as task completion time or error frequency [33].

This tutorial demonstrates the practical application of these principles throughout a complete hardware development cycle.

In this context, a novel initiative called SMARTCLOTH has been developed. SMARTCLOTH is a 3-year project funded by the Spanish 2021 Health Research Projects of the Strategic Action in Health 2017‐2020 call. It aims to develop and preliminarily test the usability of a “smart tablecloth” designed to enhance dietary management and adherence in individuals with diabetes, supporting both the portion diet system (standard in type 1 diabetes) and the plate method (recommended in type 2 diabetes). This tutorial provides step-by-step guidance on applying HCD methodology and iterative prototyping to health care hardware development, illustrated through the SMARTCLOTH project. The approach emphasizes usability testing strategies that ensure accessibility for patients or caregivers of any age and level of technological literacy, principles that can be adapted to other digital health hardware development contexts.

## Methods

### Overview

This tutorial provides step-by-step guidance on applying HCD methodology and iterative prototyping to health care hardware development, illustrated through the SMARTCLOTH project. The approach emphasizes usability testing strategies that ensure accessibility for patients or caregivers of any age and level of technological literacy, principles that can be adapted to other digital health hardware development contexts. For each phase, we describe (1) the purpose and key objectives of the phase, (2) specific tools and techniques used, (3) practical implementation details, and (4) decision-making processes when challenges arose.

We present the phases sequentially for clarity but acknowledge that in practice, iteration between phases is common and often necessary. Timelines and resource allocations are provided as reference points but will vary based on project scope and available resources.

The research team applied the DT method, following the Double Diamond model proposed by the UK Design Council [42] and adapted according to Gasca and Zaragozá [43,44]. This Double Diamond structure (Multimedia Appendix 1) identifies 4 general stages (mapping, exploring, building, and testing). In turn, the first three are subdivided into another 6. In their proposal, these authors offer specific tools, which are not mutually exclusive, to complete each stage and substage. All the tools used in the development of SMARTCLOTH are detailed in Table 1.

**Table 1.** Stages, substages, and tools in the development of SMARTCLOTH.

Stage and substage	Description	Tools
Mapping	It is used to delimit the context of the work (what is known, what is unknown, and what needs to be known) and the scope and objectives of the project.	—^a^
Mapping the team	Map the internal context of the interdisciplinary research team.	Strengths, Weaknesses, Opportunities, and Threats (SWOT) In/Out
Mapping customers	This tool helps know the profiles of patients who would benefit from using SMARTCLOTH (external context).	In-depth interviews Person Empathy map Customer journey map
Exploring	It focuses on using qualitative techniques to understand and delimit the problem. It also gathers information on what is currently being done to address it.	—
Research	It is essential to understand the problem and its related factors in depth.	Focus group (patients) Focus group (experts)
Synthesis	The specification of the problem in the design challenge, in addition to synthesizing the research into a challenge, orients the team and focuses on devising solutions on something concrete.	Design challenge
Building	It consists of embodying ideas in a prototype to define and transmit an idea quickly.	—
Devise	Through divergent thinking, the devise substage allows a broad spectrum of possible solutions to the design challenge to be explored before converging on the most effective and feasible proposals.	Brainstorming Cocreation sessions
Prototyping	It consists of materializing ideas. It is the process of quickly defining and transmitting an idea and materializing it into something physical that can be tested.	Wireframes and web prototyping 3D printing and physical prototyping
Testing	It is used to get feedback on each idea presented to end users. This stage allows us to learn what works and what doesn’t and to test functionalities without the need for the final product.	User test on the web User test on a physical prototype Usability test

a Not applicable.

### Phase 1: Mapping (January-February 2022)

#### Scope Definition and User Profiling

The key aspects of this phase include:

1. Purpose: Systematic problem assessment, team alignment, and user characterization
2. Adaptable duration: 1‐3 months depending on project scope
3. Key outputs: User profiles, project scope definition, and initial requirements
4. Application to SMARTCLOTH: According to the Double Diamond DT, the initial stage is the mapping, in which the problem is assessed, and the available resources are determined. This phase started in January 2022 and finished by the end of February 2022.

#### Team

In the team mapping phase, conducted in February 2022, we used the SWOT (Strengths, Weaknesses, Opportunities, and Threats) and IN/OUT tools to identify and categorize the team members’ strengths, weaknesses, opportunities, and threats and clearly define the elements within or outside the project’s scope. Both health care professionals and engineers participated in these sessions.

#### User and Market

Subsequently, for patient mapping, 6 in-depth interviews were conducted with health care professionals who regularly deal with patients with diabetes (4 nurses and 2 nutritionists), selected by nonrandom convenience sampling between February and March 2022. We used user-centered tools to synthesize the information collected, including “persona,” “empathy map” and “customer journey map.” The persona tool allows us to create detailed profiles of fictitious users based on actual data. Each “persona” represents a specific profile of patients who might use SMARTLOTH, detailing their demographics, behaviors, needs, and goals [45]. The “Empathy Map” was used to capture and organize information about what users think, feel, say, and do about diabetes dietary management. This tool helped us understand users’ emotions and perspectives and identify their main frustrations and desires [46]. Finally, the “Customer Journey Map” was used to map the complete journey of people with diabetes through the health care system, from the first contact to the end of their interaction. It highlighted the touchpoints, emotions, and potential barriers users face throughout their experience [47]. By identifying opportunities for improvement at each stage of the journey, we were able to define the most effective and successful functionalities of SMARTCLOTH, as well as the point in education of patients with diabetes where it could be used. Ultimately, these tools allowed us to develop detailed patient profiles, deeply understand their needs, emotions, and experiences, and comprehensively visualize their interaction with health services over time, thus facilitating the identification of critical points and opportunities for improvement that SMARTCLOTH could represent.

#### Data Analysis for Mapping

A phenomenological approach was used to analyze the data from the in-depth interviews with health care professionals specialized in diabetes education. The interviews were transcribed and meticulously reviewed. Subsequently, initial coding of the transcripts was undertaken, identifying emerging themes related to perceptions and experiences of dietary management and technology use. The codes were grouped into broad themes, such as experiences with technology, challenges in diabetes education, and recommendations for technology improvements, which are essential for identifying common patterns and developing detailed user profiles.

User-centered design tools, such as “persona,” “empathy mapping,” and “consumer journey mapping,” were developed using the information extracted from the transcripts and themes. Different user profiles were identified based on familiar patterns, and their demographic, psychographic, and target characteristics were detailed. The phenomenological approach was the most appropriate for this analysis, as it allowed capturing the subjective and emotional experiences of health care professionals and their patients, providing a comprehensive and richly detailed view that facilitated the identification of pain points and opportunities for improvement in the use of diet management hardware in patients with diabetes.

### Phase 2: Exploring (April-June 2022)

#### Research, Feasibility, and Challenge Formulation

The main aspects of this phase are as follows:

1. Purpose: Deep investigation of user needs and technical feasibility
2. Adaptable duration: 2‐4 months depending on complexity
3. Key outputs: Design challenge formulation, technical framework selection, and detailed requirements
4. Application to SMARTCLOTH: The research stage was carried out after establishing the initial set of objectives. We noticed that at least 2 research areas, clinical and technical, had to be considered.

#### Clinical Research

During the substage of the research, conducted between April and June 2022, we completed 5 focus groups to gain an in-depth understanding of the needs, experiences, barriers, and facilitators of different patient profiles regarding adherence to the recommended dietary pattern in diabetes. These included 3 focus groups with patients from the 3 profiles previously identified in the mapping stage and 2 focus groups with nurses responsible for diabetes education and management in both primary and specialized care.

#### Technical Research

We studied different software and hardware design methodologies. Hardware design requires determining the components, integrating all of them, placing each element in the device, building the device, interconnecting everything, testing it, and checking the conformance with the objectives and the experience of use of the product by different users. This task is rather long and time-consuming. Therefore, most companies do not build the final product until all the elements have been tested and approved. The users do all the testing, and in this sense, as the HCD requires large interactions with the users, it is highly suitable for combining both.

For this reason, we decide to include a quick and easy mechanism for creating a simulated device to ask users about the product without building it physically. This tool would reduce the costs of the overall design and development process, and users would provide accurate usage information for the designer. Therefore, the physical product will finally be built with the most accepted design from the users.

We analyzed several options for prototyping, including (1) creating a simulator from scratch using Python (Python Software Foundation) or other similar programming language, (2) building a mobile or tablet app, or (3) making a dynamic web page. The first option is the most powerful, as the programmer can design any behavior, but it requires much effort to develop the simulator. Moreover, adapting the simulator for every platform where it will be executed would be required. The second option has been widely used because tablets and smartphones are ubiquitous. Therefore, the app is developed once and used on lots of devices. The last option was creating a dynamic response web page. This third is the most versatile option, as any device connected to the internet with a web browser is suitable. Simulating the behavior of the SMARTCLOTH device could be like playing a game. In fact, many games are developed for HTML5-compliant web browsers, executing the code on the device and not on the server. Regarding HTML5 development, some of the most relevant game-creation frameworks are Phaser 3, Matter.js, Kiwi.js, Quintus, and CreateJS. All of them are similar, with slightly different variations and compatibilities with various web browsers and platforms. Phaser 3 is probably the most versatile HTML5 game development framework, as it has fast rendering and multitouch input capabilities on mobile and desktop browsers.

#### Synthesis

We use the design challenge tool in the synthesis substage to analyze and organize the information gathered. This tool is used to frame the core design problem and helps define the scope and objectives of the project, establishing a common starting point for the team. Typically, the challenge is formulated as an open-ended question that encourages creativity and the exploration of multiple possible solutions [48,49]. While using this tool, we focus on identifying and articulating the primary needs and desires of the users, as well as the constraints and opportunities of the project context. The formulation of the design challenge was agreed upon in a collaborative and interdisciplinary process between the health care professionals and the engineering team, trying to synthesize all the data and insights collected in the previous stages and substages.

#### Data Analysis for Mapping

Similarly, for the exploration phase, all focus group sessions with different profiles, which were audio- and video-recorded, were transcribed verbatim by a specialized company. Ricoeur’s method of hermeneutic analysis[50] a 4-stage inductive approach, was used with the support of NVivo (Lumivero) software to analyze qualitative data. First, 2 researchers reviewed the focus group data and transcripts multiple times to gain a general understanding and compile a list of main ideas. Then, the data were coded, and nodes with predefined categories were identified, establishing 13 analytical subcategories. In the third stage, new nodes were generated based on the affinity of meanings, creating 4 codes and 18 subcodes to achieve greater abstraction and discriminate the characteristics of the same idea. In the fourth stage, a greater understanding of the initial categories was achieved through the “hermeneutic arc,” considering both the initial experience and the rereading of the corpus of data. This analysis enabled us to identify which elements or characteristics act as barriers or facilitators from the perspectives of both patients and diabetes education nurses.

### Phase 3: Building (Iterative Timeline)

#### From Low-Fidelity Wireframes to Interactive Web Simulation

The relevant aspects of this phase include:

1. Purpose: Translation of requirements into tangible prototypes
2. Adaptable approach: Can use various prototyping tools based on resources
3. Key outputs: Low-fidelity wireframes, high-fidelity prototypes, and interactive simulations
4. Application to SMARTCLOTH: The team built a functional system for users to test at this stage.

#### Devise

The devise (ideation) substage was based on the implementation of brainstorming techniques and cocreation sessions and focused on the definition and integration in a single device of the functionalities that SMARTCLOTH should present to respond to the design challenge. During the brainstorming, the interdisciplinary team proposed creative and varied solutions, regardless of their initial feasibility, to maximize creativity and divergent thinking. This process allowed for exploring multiple approaches before selecting the most feasible ideas for further development [51]. The cocreation sessions also involved the interdisciplinary group to harness the diversity of thinking and experience, enrich the design process, and ensure that solutions were comprehensive and well-founded, facilitating a holistic and user-centered approach [52]. Ultimately, both sessions were characterized by interdisciplinarity, seeking solutions from the perspective of health care workers and patients but accepting the technological and time constraints for the completion of the project.

#### Prototyping

Subsequently, the prototyping substage started with the creation of low- and high-fidelity wireframes and then moved on to web prototypes. The low-fidelity wireframes were initially used to outline the basic structure and layout of the elements in our interfaces without focusing on design details. These simple, quick sketches allowed us to agilely explore different configurations and functionalities.

Finally, we developed high-fidelity wireframes, incorporating finer details, including typography, colors, and graphics, providing a more realistic representation of the final product [53,54]. Once the high-fidelity wireframes were approved, we created interactive web prototypes, which allowed us to simulate the end-user experience and evaluate the functionality and fluidity of the interface in a realistic digital environment, screen and button locations, icons, and general usability ideas. This strategy ensured the design was visually appealing, intuitive, and functional for users [22]. Phaser 3 framework enables drawing 2D objects, adding event-handling hook functions, and managing different functionalities using JavaScript. Several assumptions and simplifications had to be made. For example, we need to simulate the part of preparing the meal by humans. In real life, this process would take place at the kitchen table, by cutting portions of food or using a plate or bowl to serve each item. However, these actions fall outside the scope of the SMARTCLOTH physical device. The design will only deal with the device and its internal functioning. However, to simulate the behavior of the SMARTCLOTH virtual device, the web was split into 2 areas. On the right part, we add an area named “YOUR KITCHEN,” in which a dish and several different foods could be dragged to place the dish above the balance or add or remove pieces of food to the dish or from the dish, or even directly above the weight. As a simplification, 2 buttons in the “YOUR KITCHEN” area were added to add 25 g of a given previously activated food or to remove 25 g from the piece of food. The piece of food grows bigger or smaller according to the number of 25 g clicks the user has pressed. If the piece of food goes to 0 g, it disappears from “YOUR KITCHEN.” We decided that the “YOUR KITCHEN” area should be placed on the left part of the screen. On the right side of the screen, the SMARTCLOTH device was simulated.

**Figure 1.**
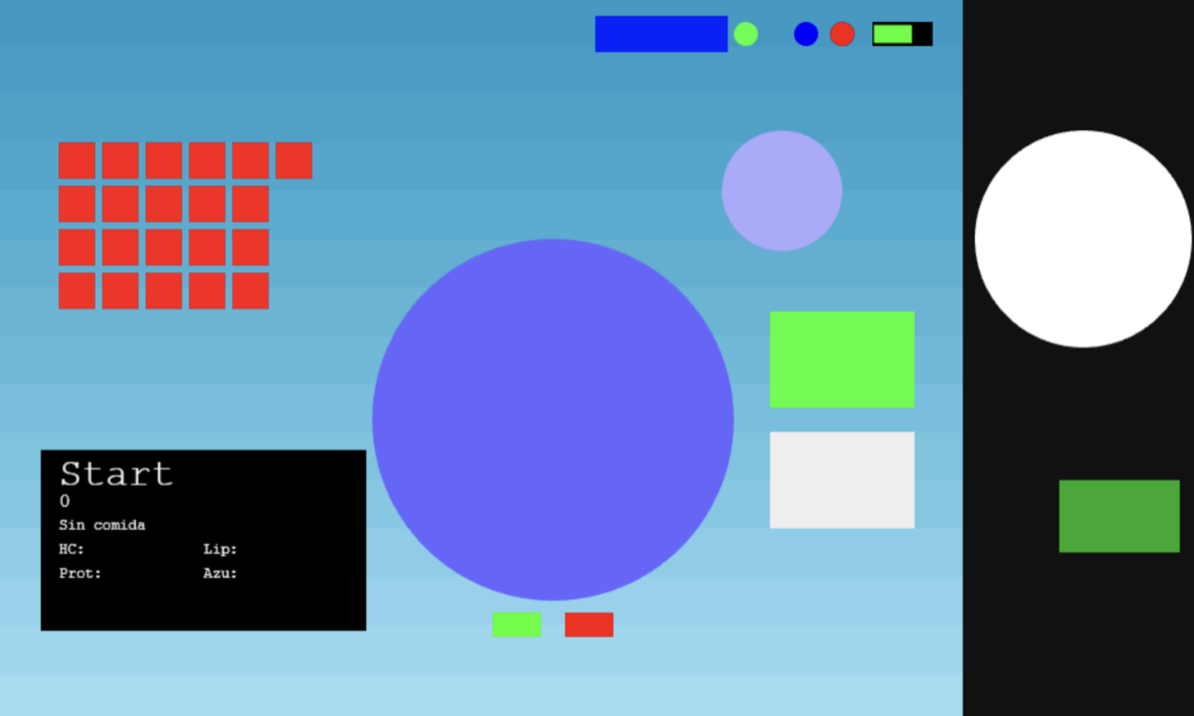
SMARTCLOTH initial web prototype – not shown to users, internal testing only.

The SMARTCLOTH web prototype was designed and developed using the Phaser 3 JavaScript framework. The initial prototype, shown in Figure 1, was created using simple forms (circles and rectangles). Members of the project team tested this prototype internally to check the conceptual functionality of the elements.

### Phase 4: Testing (October-December 2022)

#### Iterative Usability Evaluation and Refinement

The main elements of this phase include:

1. Purpose: Iterative usability evaluation and refinement
2. Adaptable cycles: 2‐5 testing iterations recommended
3. Key outputs: Validated design, usability metrics, user feedback, and refined prototype
4. Application to SMARTCLOTH: SMARTCLOTH web prototypes designed in the previous phase were used for the testing stage. Five testing sessions were conducted between October and December 2022 (Multimedia Appendix 2), involving 7 patients with diabetes representing the 3 previously identified profiles. Each prototype was tested by users and supervised by project team members. During these sessions, participants performed a series of specific tasks, detailed in Table 2, to evaluate the functionality and usability of the prototype. Each test was recorded with a video camera so that their behavior could be checked in the future. The users provided some annotated feedback, which was used to make all the changes for the following prototype evolution.

**Table 2.** Tasks applied in the SMARTCLOTH user tests.

Test	Elements involved	Difficulty
Weigh one portion of grilled salmon (300 g)	Dish Sliced salmon	Easy
Weigh a 150 g apple	Apple	Easy
Weigh a plate of 150 g of pasta, 50 g of tomato, and 50 g of canned tuna.	Dish Pasta (boiled)^a^ Tomato, natural Canned tuna (raw)	Moderate
Prepare a chickpea salad and a plate of 150 g of pasta, 50 g of tomato, and 50 g of canned tuna.	Chickpeas (boiled) Tomato, natural Canned tuna (raw)	Moderate
Simulate two meals on the same day: Breakfast: 150 g whole milk + 100 g whole wheat bread + 50 g butter Lunch: 200 grams of boiled zucchini + 50 grams of oil + 150 g of breast + 1 boiled egg + 150 grams of banana	Glass Plate Whole milk Butter Wholemeal bread Dish^b^ Courgette (boiled) Olive oil Breast Hard-boiled egg Banana	Hard

a SMARTCLOTH can differentiate between raw and boiled foods.

b When having 2 meals, 2 dishes are needed.

The think-aloud technique was used to collect qualitative data on the user experience during the development of the tests. This technique involves participants verbalizing their thoughts, emotions, and actions as they interact with the prototype, providing insight into their cognitive process and identifying usability issues [55,56]. The choice of the think-aloud technique, widely recognized in usability research, was based on its ability to reveal critical aspects of user behavior that would not be evident through other evaluation methods [57]. The data collected was analyzed to identify common patterns, recurring problems, and SMARTCLOTH usability, providing the basis for subsequent design iterations and ensuring that the final product was intuitive and functional for end users.

We designed a set of questions for the users after each test. Each user was asked about how many kilocalories that meal had, how many rations of carbohydrates that meal had, and some other similar questions. We took note of the errors they committed and the overall interaction with the simulator. Finally, we asked them to express their feelings and suggestions about using that simulator (Multimedia Appendix 3).

For each recorded test, we wrote down several direct data points extracted from the videos, including number of attempts for each test, total time to complete the test, best time try, average time of the attempts, number of errors, number of explanations and positive reinforcements, and stops in the attempts (direct instructions and indirect indications). Some other indirect data were extracted from the videos of the answers after the test, including responses completed without help, with help, and unanswered or incorrectly answered. In the following, we describe every term extracted from the videos:

1. Attempt: Time interval in which the user works with the prototype to accomplish the proposed task.
2. Explanation: Time interval in which the user receives a more or less detailed explanation of the task, operation of the device, or some other information related to the experiment.
3. Error: Detection of malfunctioning of the prototype. Depending on its severity, it can generate a restart of the attempt.
4. Positive reinforcement: Affirmative expression confirming to the user that he or she is proceeding correctly.
5. Direct help: Explicit indication of the next step to follow.
6. Indirect help: Implicit indication, usually in the form of a question, to give the user clues on how to continue.

In each test, we showed the SMARTCLOTH web prototype on a large (50 inches) screen connected to a computer with an internet connection. We explained every button and element to users, describing their use and behavior. After that, one by one, each user was asked to manage different types of meals with increasing complexity. Each user entered the room where the test was carried out at a given time without interaction with the rest of the users so that no bias could arise when exchanging information among them.

#### Data Analysis for Testing

A qualitative approach based on content analysis was used to analyze the data obtained through the think-aloud methodology and the annotations made during testing. The sessions were also videotaped, transcribed, and coded. User comments and behaviors were analyzed and grouped into categories related to usability, understanding of the hardware, difficulties encountered, and suggestions for improvement.

### Positionality Statement

The research team comprised health care professionals (nurses and nutritionists) and engineers (computer and electronic engineering) affiliated with academic and biomedical research institutions in Spain. Our backgrounds combine expertise in diabetes education, nutrition therapy, digital health innovation, and HCD methodologies. Several members have extensive clinical experience supporting individuals with diabetes and are committed to enhancing patient education and dietary adherence. Others contribute technical expertise in prototyping, usability, and interactive system design.

We acknowledge that our values—particularly a belief in the potential of technology to enhance lifestyle management and our commitment to patient-centered care—have influenced the research process. These perspectives informed our emphasis on usability, patient engagement, and contextual adaptation of SMARTCLOTH. At the same time, we recognize that our orientation toward health care innovation could predispose us to highlight the positive aspects of digital interventions.

To address these influences, we used reflexive practices, triangulated perspectives within an interdisciplinary team, and integrated continuous feedback from patients and health care professionals throughout the design and testing process. This collaborative and iterative approach aimed to ensure that the findings reflect not only our assumptions but also the lived experiences and challenges of people with diabetes.

### Ethical Considerations

The project complied with current Spanish regulations on bioethical research, personal data protection, and bioethics, following Law 3/Dec 5, 2018. In addition, the fundamental principles of the Declaration of Helsinki (1964), the Council of Europe Convention on Human Rights and Biomedicine (1997), and the United Nations Educational, Scientific and Cultural Organization (UNESCO) Universal Declaration on the Human Genome and Human Rights (1997) were respected. The research was approved by the Ethics and Research Committee of Cordoba (Act n° 273, ref. 3754). Since video and audio recordings were made of the participants, they were also asked to sign a consent for transferring image rights. In the case of minors, these consents were signed by their parents.

This study was conducted in accordance with ethical standards on privacy and confidentiality. No identifying information of participants is included in the manuscript or supplementary materials, and all potentially identifiable data were omitted unless scientifically essential. All participants signed informed consent and were able to withdraw it at any time if they considered it appropriate; in the case of minors, informed consent forms were signed by their parents. Since video and audio recordings were made of the participants, they were also asked to sign a consent form for transferring image rights. No compensation was provided to participants.

## Results

### Methodological Insights and Case Example Outcomes

This section presents the outcomes of applying the Double Diamond methodology to SMARTCLOTH development, organized to highlight both the specific results obtained in our case example and the generalizable methodological insights that emerged from the process. We emphasize decision points, adaptations, and lessons learned that can inform other hardware development projects.

### Mapping

#### Team Mapping

The team considered that the main threats identified included the recent pandemic (and its long-term consequences), the difficulty in changing dietary habits, and the increasing number of similar products on the market. Significant weaknesses included limited time availability and lack of human resources, the disparity of interests among the researchers, and the fact that they were involved in several projects (multitasking). However, the project had important strengths, such as skills in web technologies, support from various institutions, and a strong motivation for health education. Notable opportunities included (1) direct access to patients and diabetes care professionals, (2) a positive view of technology in the wake of the COVID-19 pandemic, (3) the availability of funding, and (4) the opportunity to apply to different national calls for hiring professionals to reinforce the team, all of which supported the feasibility and potential success of the project.

In addition, it was crucial to determine which technological challenges could be addressed by the team. Among the functionalities of SMARTCLOTH that the team felt they were capable of developing were food weighing, barcode reading, design and physical printing of prototypes, design of buttons for user interactions, estimation of calories and macronutrients ingested at each meal with the ability to make longitudinal records of intake, and a website where each user could submit and consult this information, among others. However, it was decided that other functionalities (shopping list, voice recognition, and offering feedback on the diet quality) exceeded the time and economic capabilities of the equipment. A graphical summary of the results of these dynamics is available in Multimedia Appendix 4.

#### User Profiles Mapping

In the in-depth interviews conducted to identify user profiles, 6 professionals (4 diabetes nurse educators and 2 nutritionists) with varying experience in diabetes management but all over 3 years participated. The analysis of these interviews helped us identify potential users of SMARTCLOTH, identifying 3 profiles.

The first profile (given the name Kevin) is an adolescent with T1DM. Kevin manages his insulin well and understands his condition, but he faces challenges related to social life and eating out. He represents teenagers who think changing their diet is complicated and do not want to be the “weirdo” among their friends. They perceive their health as out of control and sometimes feel frustrated because they cannot eat like everyone else. These users seek to enjoy and have fun while eating but face occasional family conflicts and struggle to adjust their eating habits. In their journey as users, they face (together with their family) initial doubts and fears, mistrust and loneliness, but show interest and confidence in seeking new tools to manage their condition.

The second profile, Julia, represents mature adults (between 50 and 65 years old) with T2DM. This profile corresponds to active workers, which makes it difficult for them to maintain a regular diet due to their schedules. They are motivated to improve their dietary knowledge and are concerned about their health but find it difficult to plan and prepare healthy meals. Overall, they maintain acceptable control of their disease. However, they perceive diet as a significant sacrifice and feel uncertainty and decreasing motivation when faced with limited consultation time and the confusion of scattered and, in many cases, contrary information they can find on other media, such as the internet. They often commit dietary transgressions linked to family gatherings, parties, and work shifts. In their journey as users, they experience an internal struggle to follow the diet and improve their eating habits while seeking to maintain an active social life and enjoy food with family and friends.

The third profile (Paco) corresponds to older people (aged more than 65 years) with T2DM and low adherence to dietary treatment. Generally speaking, they have a long history of the disease and face insecurities and a lack of interest in following the diet. In addition, they have no objection to adhering to pharmacological treatment, although they say that insulin injections frighten them a little. They prefer traditional food and do not want to give up what they enjoy. This group of users thinks it is difficult to change their eating habits at their age, and they feel distrustful and uncertain about the effectiveness of the diet. In the case of men, it is common for them to delegate responsibility for their diet to their wives (which can lead to family conflicts), and in general, they have not changed their lifestyle and do not follow the recommended diet. In their journey as users, this resistance to change stands out; they argue with their partners about meals and feel overwhelmed by the amount of information about diet despite understanding the importance of taking care of themselves. SMARTCLOTH could be a solution for this group, provided they are motivated. However, the fact that they are not motivated and have poor disease control makes it unlikely that they will ultimately be users of this device. These profiles helped design SMARTCLOTH to meet the specific needs of each user group, providing solutions tailored to their particular contexts and challenges.

Multimedia Appendix 5 (in Spanish) summarizes the tools used to define these profiles (Persona, Empathy Map, and Consumer Journey Map).

#### Exploring

Once these 3 profiles were identified, 5 focus groups were conducted, 1 for each profile, and 2 other groups that included primary and specialized care professionals responsible for diabetes education. The characteristics of the participants are detailed in Table 3 for the patients and Table 4 for the nurses.

**Table 3.** Descriptive variables of the patients participating in the focus groups.

Code	Age (years)	Sex	Marital status	Area	Employment status	Educational level	Socioeconomic status	Diagnosis DM^a^ (year)
Patients with T2DM^b^								
T2DMP1	71	F^c^	MR^d^	U^e^	HW^f^	P^g^	ID^h^	2018
T2DMP2	65	F	MR	U	RT^i^	P	IE2^j^	2007
T2DMP3	70	F	MR	U	HW	P	IE1^k^	2004
T2DMP4	77	F	MR	U	RT	P	IE1	2006
T2DMP5	61	F	MR	U	AJS^l^	P	IE1	2009
T2DMP6	68	M^m^	MR	U	RT	P	IE1	—^n^
T2DMP7	67	M	MR	U	RT	P	ID	2012
T2DMP8	53	F	MR	U	A^o^	Sec^p^	IE1	2018
Patients with T1DM^q^ and parents								
T1DMP1	16	F	SN^r^	R^s^	ST^t^	Sec	ID	2014
T1DMP2	17	M	SN	U	ST	Sec	ID	2014
T1DMP3	15	M	SN	U	ST	Sec	IA1^u^	2019
T1DMP4	15	M	SN	U	ST	Sec	IC^v^	2018
PART1DMP1^w^	55	M	MR	R	A	P	ID	—
PART1DMP2	50	F	MR	R	HW	P	ID	—
PART1DMP3	45	F	SD^x^	U	A	S	ID	—
PART1DMP4	48	F	MR	U	A	US^y^	IA1	—
PART1DMP5	46	F	MR	R	HW	P	IC	—
PART1DMP6	45	M	MR	R	A	Sec	IC	—

a DM: diabetes mellitus.

b T2DM: type 2 diabetes mellitus.

c F: female.

d MR: married.

e U: urban.

f HW: housewife.

g P: primary.

h ID: 1313‐1602€/month (1€=US $1.16).

i RT: retired.

j IE2: less than 745€/month.

k IE1: 745‐1312€/month.

l AJS: active job search.

m M: male.

n Not available.

o A: active

p S: secondary.

q T1DM: type 1 diabetes mellitus.

r SN: single.

s R: rural.

t ST: student.

u IA1: more than 3005€/month.

v IC: 1603‐2145€/month.

w FAMXX: A family member or primary caregiver of a patient with type 1 diabetes mellitus.

x SD: separated or divorced.

y US: university studies.

z PAR: parents.

**Table 4.** Descriptive variables of the diabetes nurse educators participating in the focus groups.

Code	Age (years)	Sex	Educational level	Socioeconomic status	Years of experience in diabetes education
DNE1^a^	42	F^b^	U^c^	IB^d^	16 (as a primary nurse, although in health centers, they are not exclusively dedicated to this).
DNE 2	55	F	U	IB	17 (as a primary nurse, although in health centers, they are not exclusively dedicated to this). She was diagnosed with diabetes 30 years ago.
DNE 3	58	F	U	IB	32 (as a primary nurse, although in health centers, they are not exclusively dedicated to this).
DNE 4	55	F	U	IB	6 years as a diabetes nurse educator in the endocrinology department.
DNE 5	55	F	U	IB	6 years as a diabetes nurse educator in the endocrinology department.
DNE6	59	F	U	IB	4 years as a diabetes nurse educator in the endocrinology department.

a DNE: diabetes nurse educators.

b F: female.

c U: university studies.

d IB: 2146‐2451€/month (1€=US $1.16).

Patients with T1DM and their caregivers (fathers, mothers, or both) indicated that diabetes management is more difficult in situations outside the daily routine, such as when eating out or at social events. They also mentioned that a lack of knowledge and confidence in portion measurement and carbohydrate counting can be a significant barrier. Other factors highlighted were the influence of the social environment and the need for flexibility in dietary choices. However, motivations for maintaining adherence included the desire for future well-being and effective diabetes management to avoid long-term complications. Possible functionalities we extracted for this group included food scanning and carbohydrate counting, personalized alerts and reminders, a healthy recipe database, real-time monitoring and feedback on glucose levels, adjusting dietary recommendations based on current readings, compatibility with glucose sensors, and educational and motivational functionalities.

Participants belonging to profile 2 (with T2DM and with acceptable metabolic control) highlighted that diabetes management is affected by factors, such as the social environment, the availability of healthy food at home, and the lack of structured support to follow a proper diet. Many mentioned that they find it difficult to follow dietary recommendations due to the temptation to consume unhealthy foods readily available in their environment. In addition, some patients expressed that the lack of clear and personalized information about recommended diets complicates their adherence. On the other hand, motivations for maintaining adherence include avoiding major complications and maintaining a good quality of life. Functionalities for this group included personalized dietary recommendations with personalized meal plans, food scanning and analysis, reminders and alerts (for food intake, medication, and glucose level monitoring), interactive nutrition education, connection to monitoring devices, community and motivational support (creating a community support platform to share experiences and advice, and receive support), and physical activity recording.

Finally, from the group of patients belonging to profile 3, participants mentioned the difficulty of following a diet due to the constant temptation to consume unhealthy foods available at home, the lack of social and motivational support, and the perception that measuring and weighing food is tedious and difficult to maintain in the long term. Some patients expressed that physical exercise was used to justify consuming nonrecommended foods, believing that physical activity would compensate for dietary excesses. Lack of consistency and additional health problems were also important barriers. On the other hand, the main motivation for following the diet was to improve quality of life and avoid serious health complications. However, there was little motivation to adhere to the recommended dietary pattern. Continuous monitoring of glucose levels, recording of physical activity, alerts and reminders, nutritional and motivational education, food scanning, and carbohydrate counting were also highlighted as functionalities for managing their disease.

According to specialist care nurses for patients with diabetes, patients with T1DM are often newcomers, those who are newly diagnosed, or those with years of evolution who require ongoing education on the management of their condition, including insulin administration and carbohydrate counting. On the other hand, patients with T2DM, especially those poorly controlled (profile 3), tend to be more resistant to changing habits and face additional complications that make adherence to dietary guidelines complex and are the reason for referral from primary care. According to these nurses, the main barrier for patients with T1DM is the lack of adequate initial education, especially at the time of diagnosis, when both patients and their families feel overwhelmed. Adolescents with T1DM (profile 1) sometimes have low adherence to dietary recommendations due to rebelliousness and preference for unhealthy foods. In addition, constant carbohydrate counting and weighing is tedious for many, which can lead to errors in insulin management. However, structured and ongoing diabetes education is a crucial facilitator, as are family support and the use of technological tools, such as mobile apps and continuous glucose monitoring devices, which help to improve adherence. For patients with T2DM, the most prominent barriers include ingrained eating habits and the perception that following a diabetic diet is complicated and tedious. Cultural and social factors also play an important role in dietary adherence. Many patients prioritize medication over diet, which hinders adequate glycemic control. On the other hand, facilitators for these patients include ongoing education and awareness of the importance of diet in diabetes management. Social and family support can motivate patients to follow an appropriate diet, and using personalized meal plan tools is also beneficial.

As a result, the specialized care nurses believe that SMARTCLOTH could include functionalities such as the implementation of a food scanning and analysis system that provides detailed nutritional information that would help patients with carbohydrate counting, personalized meal plans, and detailed menus to avoid the monotony and perceived complication of following a diabetic diet, and automatic alerts and reminders for taking medications, meals, and glucose measurements. In addition, they comment that mobile apps that help to calculate portions and adjust insulin intake according to the meal would be a valuable tool, as well as interactive and ongoing nutrition education, including modules on nutrition and diabetes management, which can improve knowledge and adherence.

Primary care nurses mainly care for patients with T2DM and, to a lesser extent, patients with T1DM, with one of the participating nurses having T1DM, which brought a unique perspective to the session. According to the professionals, patients with T1DM (profile 1) often arrive well educated and controlled from diagnosis, especially if they debut in childhood, but face barriers such as the perception of diet as tedious and lack of adequate initial education. At the same time, facilitators include good diabetes education and family support and engagement. Patients with T2DM face barriers, such as ingrained dietary habits, cultural influences, and a passive attitude toward diabetes, with facilitators including continuing education, social support, and the use of practical tools. For all these reasons, they agree with the other nurses that functionalities, such as food scanning, personalized meal plans, automatic alerts and reminders, carbohydrate counting apps, interactive nutrition education, and a community support platform could improve dietary adherence.

### Building

The qualitative assessment of patients’ perspectives revealed a set of needs that were systematically translated into essential functionalities, which necessarily had to be incorporated into the SMARTCLOTH device. This process ensured that the design responded directly to real user requirements, providing core features, such as real-time conversion of food weights into macronutrient portions, integrated calorie estimation, food group identification, and barcode or QR code scanning (Table 5). However, other needs could not be satisfied due to technical constraints, challenges encountered during the prototyping phase, or because they exceeded the project’s scope and objectives. These unmet needs, also documented in Table 5, included, for example, the provision of insulin dosing guidelines, auditory feedback when pressing keys, and additional nutritional recommendations, such as specific weight-loss or lactose-free diets. All this information was subsequently transferred to a SMARTCLOTH simulation website that supported the first round of user testing.

**Table 5.** Identified needs and functionalities developed to meet them^a^.

Need identified	Functionality developed to respond to the need
Difficulty converting food weight in grams into macronutrient portions. Lengthy calculations, leading to disengagement and discontinuation. Uncertainty regarding carbohydrate intake when estimating the insulin dose to be administered (T1DM)	The screen provides real-time display of both weight in grams and the corresponding macronutrient portions.
Need to rely on multiple devices (scales, calculators, plates, depending on the food) to calculate each dish.	SMARTCLOTH integrates all required tools into a single device.
Lack of information on caloric intake (particularly relevant for some patients with T2DM and obesity)	The screen provides real-time estimation of the caloric content of each dish.
Lack of knowledge and difficulty identifying foods, the group they belong to, and their main macronutrient	SMARTCLOTH includes a panel of 20 food groups illustrated with images; selecting a group displays a list of foods within it.
Difficulty estimating macronutrient portions in packaged foods.	SMARTCLOTH incorporates barcode and QR code scanning.
Lack of overview of daily and weekly caloric and macronutrient intake.	SMARTCLOTH stores consumption data, accessible via a web platform.

a Needs that have not been met*: *(1) guidelines for insulin dosing based on carbohydrate intake; (2) auditory feedback when pressing device keys; (3) provision of healthy recipes or meal plans; (4) additional nutritional recommendations (eg, weight-loss and lactose-free diets); (5) touchscreen interface (requested primarily by younger patients).

The SMARTCLOTH web prototype had 15 internal versions, which provided 3 prototypes to be tested by the users. All the versions were tested internally to check whether the system behaved correctly. After those internal prototypes, users tested each new version and modified it accordingly. Main modifications came from the clinical part as new meals were designed, and thus, the “YOUR KITCHEN” had to be altered to add and remove food to create the meal.

Some other modifications were required because of the users’ feedback about the position or size of the elements (screen, balance, or buttons). These modifications were the most interesting from the design point of view, as they provided firsthand feedback from the users’ usage experience.

Besides, the researchers performed express modification sprints during testing sessions to change some behaviors or correct minor errors that would not affect the rest of the users in the testing. Some of these modifications were done in less than 10 minutes, from detecting the requirement to be modified or added to uploading the new code to the web server. These modifications could be done in real-time as the modified file was on the web server. Therefore, each new prototype version was available just after reloading the web page.

**Figure 2.**
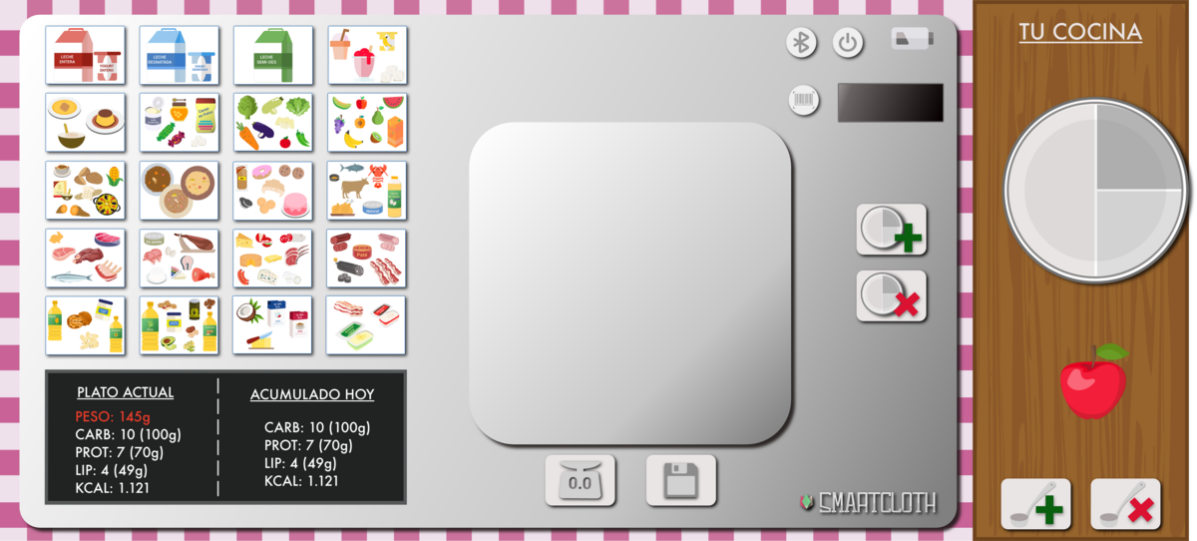
SMARTCLOTH’s first web prototype shown to users.

The following version, shown in Figure 2, represented a significant change. All the simple forms were substituted by images representing real buttons, screens, etc, so users could feel the usage experience as real as possible.

All the users in the test were surprised about the SMARTCLOTH prototype. They all liked it very much and were committed to helping in the test the best they could. We noticed that the users did not “read” the screen. The screen’s position in the lower-left corner of the SMARTCLOTH prototype was not the best choice. All the users expected it in the upper left corner. They did not recognize most foods in the buttons representing food groups. The quality or size of those buttons did not allow the users to understand the foods included in every food group well. Some of them suggested adding examples on the screen showing the foods included in each group. Some of them did not like the position of the buttons for adding a new dish or removing a dish from the meal. They preferred all the control buttons related to the meals to be in the same area. The older users asked us to add text to the control buttons. Therefore, we added “TARA” (in Spanish) to the tare button.

**Figure 3.**
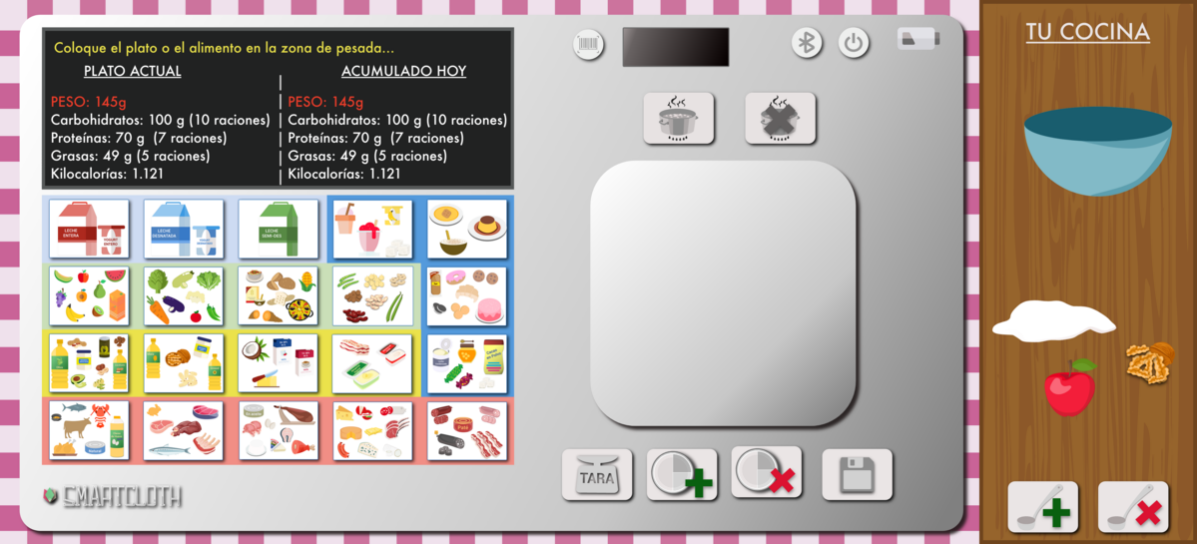
The second evolution of the SMARTCLOTH web prototype shown to users.

The second version of the SMARTCLOTH web prototype, shown in Figure 3, changed the composition. As suggested in the previous test, the screen and the group of food buttons were swapped. Besides, we added new buttons related to cooked or raw foods. The clinical team requested this for the project. We decided to move the add dish and remove dish buttons below the weight, beside the Tare and Save buttons, as indicated in the previous test. Those two new buttons (cooked and raw) were placed on top of the weight. We added text inside the buttons describing the functionality of each of them, as mentioned in the previous test.

In the second evolution test, we noticed that the Tare button was useless in how users interacted with the prototype. The system did the tare when a new group of food buttons was pressed. Therefore, we removed the Tare button and the necessity of pressing 2 buttons.

**Figure 4.**
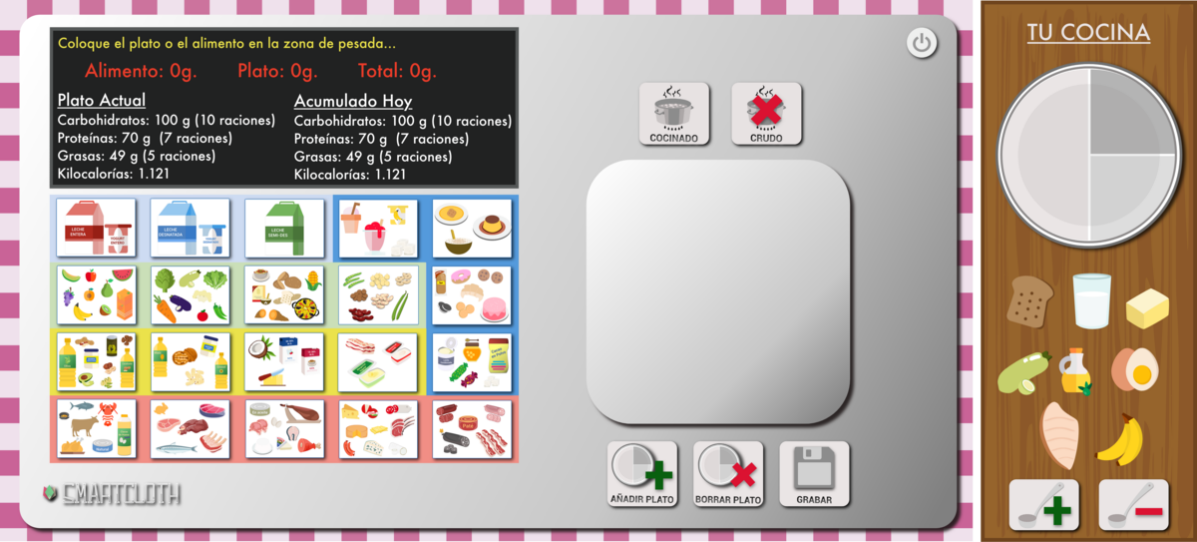
Final testing of SMARTCLOTH HTML5-compliant web prototype.

Besides, users asked us to remove them from the final prototype, as neither barcode nor Bluetooth functionalities were included. In the same way, as the power consumption was not simulated in the web prototype, the battery icon was removed in the SMARTCLOTH final prototype. All these changes led us to the final SMARTCLOTH web prototype, shown in Figure 4.

#### Testing

Three sessions were carried out. Every session was recorded and analyzed afterwards.

##### Tests 1 and 2

The SMARTCLOTH first web prototype, shown in Figure 2, was used in tests 1 and 2. The first test was a meal of 150 g of apple, and the second was 300 g of salmon. The analysis of the recorded video provided the results shown in Multimedia Appendix 6. Each user was anonymized by the acronym profile (P1/P2/P3) and user (1-5). For instance, P1.3 represents user 3 of profile 1 (T1DM).

##### Tests 3 and 4

The second version of the SMARTCLOTH web prototype, shown in Figure 3, was used in tests 3 and 4. Test 3 consisted of 150 g of apple, 250 g of yogurt, and 100 g of nuts. Test 4 consisted of 150 g of pasta, 50 g of tomato sauce, and 50 g of tuna. The analysis of the recorded video provided the results shown in Multimedia Appendix 7.

##### Test 5

The final version of the SMARTCLOTH web prototype, shown in Figure 4, was used in test 5, a complete meal composed of 3 dishes. The analysis of the recorded video provided the results shown in Multimedia Appendix 8.

**Figure 5.**
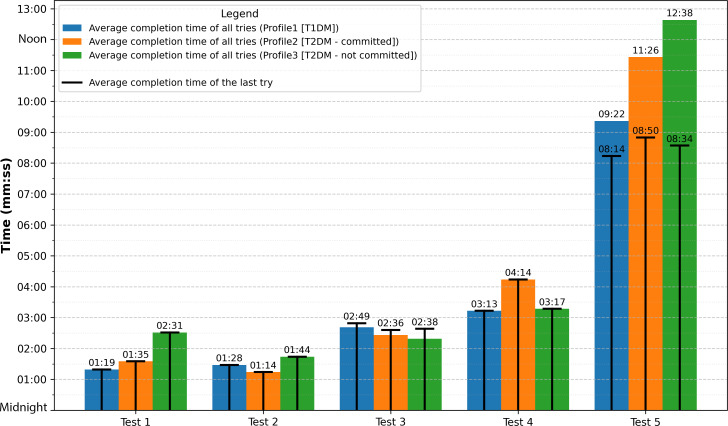
Average completion times for user tests. Bars represent the average time of the tries for each test and user type, while the lines represent the average time of the last try for each test and user type. T1DM: type 1 diabetes mellitus; T2DM: type 2 diabetes mellitus.

Figure 5 shows the average time of the last try and the average time of the tries for each test, according to user profiles.

## Discussion

### Principal Findings

This tutorial has demonstrated the systematic application of HCD and DT methodology to develop digital health hardware, using SMARTCLOTH as an illustrative case example.

This DT methodology is widely used to create technological solutions for chronic health problems. For example, the study by Petersen and Hempler et al [58] used it to develop a mobile app that helps patients with diabetes manage their disease more effectively. The researchers found that the app significantly improved glycemic control and adherence to treatment. Another study used this methodology to design a telemedicine platform for patients with hypertension, resulting in a marked improvement in blood pressure monitoring and patient satisfaction [59]. Like our work, both studies showed optimal results, highlighting the effectiveness of DT in developing technological solutions for managing chronic diseases, where treatment adherence has been shown to be a key element.

### Reflections on the HCD Process

#### Mapping

##### Team Mapping

Concerning the research team’s preassessment, despite the threats and weaknesses identified, this technique offered an interesting approach for researchers to maximize their strengths and take advantage of the opportunities available. The recent pandemic presented significant challenges, coupled with the difficulty in changing dietary habits and competition with similar products on the market. In this sense, an honest and strategic assessment of the project’s human, temporal, and economic capacities beforehand allowed the development of all stages of the DT methodology to be adjusted. Despite this, a dearth of studies uses or reports the results of these preassessment strategies. However, the orientation and focus that originated in the team underlines the need for robust methods to identify the potential of interdisciplinary groups and concretize the work in technological projects to improve adherence to lifestyles in chronic diseases. Recent studies have highlighted similar challenges and proposed similar solutions to overcome identified weaknesses in health-related technology projects [60-62].

##### User Profiles Mapping

Using end-user-centered design tools, such as “persona,” “empathy mapping,” and “customer journey mapping” has been particularly interesting. Traditionally used in business and the private sector to improve the benefits of business propositions, these tools are applied novelly in the health sector. However, this approach has been evidenced to help increase usability and adherence to use by patients with chronic conditions. For example, a recent study used the persona tool to design a medication-tracking app for patients with cardiovascular disease, significantly improving treatment adherence [63]. Another study implemented the “customer journey map” in developing a telerehabilitation program for patients with chronic obstructive pulmonary disease, finding that the tool helped identify friction points and improve the user experience, thereby increasing patient engagement and satisfaction [64]. Similarly, using the “empathy map” in developing a support platform for patients with diabetes allowed developers to better understand users’ emotional and functional needs, leading to a more intuitive and effective design [65]. Like the studies discussed above, our work has also found these tools very useful in understanding and segmenting our users, having identified three profiles (1) First profile comprised one of adolescents and their caregivers with T1DM, who manage their insulin well but face social and dietary challenges; (2) a second profile, of mature adults with T2DM who, while having difficulty maintaining a diet, try to engage with treatment; and (3) a third profile, corresponding to older people with T2DM and with low adherence to treatment and severe difficulties in modifying lifestyles.

Comparing these profiles with those reported in the literature, we found important similarities and differences. For example, a study on medication adherence in patients with diabetes identified that levels of family support play a crucial role in adherence to treatment. Patients who perceive greater instrumental support from family members, such as help with diet and medical appointments, tend to show better adherence to treatment regimens [66,67]. This finding aligns with our second profile, where family support is a key facilitator. Another study highlighted that barriers, such as lack of knowledge about diabetes and unsupportive family behaviors, such as negative interference with medication adherence, are significantly associated with poorer glycemic control outcomes [68]. This finding is particularly relevant for our third profile of older people with T2DM, who often have difficulty following dietary recommendations due to ingrained dietary habits and lack of structured support [69].

In addition, implementing continuous quality improvement programs in diabetes management has been shown to improve treatment adherence, promote self-care, and increase patients’ knowledge about their disease [66,70]. This strategy could benefit all the profiles identified in our study, providing a constant support structure and ongoing education.

Ultimately, these tools have allowed us to identify each group’s specific needs and barriers, facilitating the design of the functionalities that SMARTCLOTH should present as the most effective and personalized solutions to improve adherence and dietary management of diabetes. These were the key issues addressed in the next stage.

### Exploring

A determining factor in the design of a technological solution is its adaptation to the needs of the end users. In this sense, during the exploring stage, factors that facilitate or hinder adherence to a diet that helps glycemic control in patients with diabetes were detected. At this point, we emphasized those identified barriers or facilitators relevant to the design of the proposed technological solution.

Among the 3 profiles identified, the environment (social events, eating outside the home, family pressure, constant exposure to unhealthy foods...) appears as a common element that gets in the way of following an adequate diet, something described in studies carried out in other countries [71,72]. This situation makes glycemic control difficult, either by eating high-calorie meals, high consumption of proteins and/or fats that alter glucose absorption, or difficulty counting carbohydrates ingested [73,74].

Of the elements mentioned in the previous paragraph, the high availability and exposure to high-calorie foods, unhealthy fats, and free sugars, especially ultraprocessed foods, are particularly important today. Studies show that consumption of these foods is very high [75], so it must be assumed that SMARTCLOTH users will incorporate them into their diet. In this context, a barcode scanner was envisaged to monitor the intake of these products accurately and to increase control and awareness of what is involved in eating them [76].

On the other hand, the difficulty in counting carbohydrates is not only limited to social life but also at specific times (the first months after diagnosis of the disease, adolescence, after years with the disease...), there is insecurity and/or relaxation due to lack of training, in the first case, or fatigue due to the tediousness of the process required to do so, in the second [77,78]. The counting of this macronutrient, especially in patients with T1DM, is fundamental in glycemic control, as it defines the insulin dose [79]. Therefore, the mainstay of SMARTCLOTH is the calculation of grams and servings of the 3 immediate principles and the monitoring of intakes throughout the day. Farooqi et al [80] found that this type of functionality is highly relevant in diabetes management and in increasing adherence to the necessary therapeutic approaches, as also found by other authors [81-83].

Furthermore, the ability to structure an adequate diet is an important limitation for most of the population. During the focus groups, patients with T2DM openly expressed this problem [71,84]. This difficulty is a major problem, as the correct dietary therapeutic approach (quality food, reduction of free sugars, moderate carbohydrate diet, and reduction of caloric intake) is essential to improve metabolic control [85]. In this context, SMARTCLOTH presents itself as an ideal tool for dietary management. In addition to the aforementioned functions, the digital tablecloth has to allow the monitoring of caloric intake at each meal and accumulated caloric intake over a day, which will allow moderation of energy intake and loss of body fat, which has emerged as the main goal set by health care workers for patients with T2DM [86]. However, SMARTCLOTH should be accompanied by dietary recommendations made by qualified personnel tailored to the individual patient’s needs.

A lack of culinary knowledge also limits the ability to structure and follow an appropriate diet [87,88]. This shortage means that most meals are not organoleptically comparable to those offered by the food industry and require more preparation time [89,90]. Therefore, taking advantage of the barcode reader, the researchers decided to design a book and a database of standardized healthy recipes that would (1) improve the quality of meals, thanks to a better choice of foods; (2) reduce the time spent in the kitchen, through simple recipes; (3) increase the palatability of meals, through the introduction of very tasty recipes; and (4) reduce the difficulty of controlling meals, because the simple reading of the barcode associated with the recipe will allow the portion to be weighed directly without the need to consider the ingredients separately.

Monitoring blood glucose and its coupling with dietary intake is of particular concern to health care staff caring for this group of patients [91-93]. In this case, SMARTCLOTH will upload all intakes recorded on the device to a database. This information will be uploaded to a website, allowing the patient to enter their blood glucose and graphically observe the correlation between blood glucose and intake. This functionality can help to establish a pattern or detect errors in the patient’s self-care and help to intervene more precisely.

Finally, it should be noted that although SMARTCLOTH is designed and developed according to the needs expressed by patients, motivation will play a key role in its use, as, like any intervention, it will require commitment from users [94-96]. As evidenced throughout this research, it is unlikely that those who do not want to change their habits (profile 3) are likely to do so.

Undoubtedly, SMARTCLOTH will not address all the barriers that were identified, especially some related to the environment. This is because the final prototype will not be of a size that allows it to be used outside the home. However, a solution may be offered by future iterations of SMARTCLOTH, which may be more portable so that it can be used outside the home.

### Building

Initially, the prototype was planned to be developed physically. However, adopting the web-based implementation strategy has greatly benefited the project. Web technologies have made it possible to speed up prototyping and make changes at no economic cost.

Three significant evolutions of the prototype (with multiple small internal modifications) have occurred. Agile methodologies guided these evolutions, and mini prototypes were rapidly developed. The development team tested these internally, and if they passed the quality standards, they were integrated into the prototype to be tested by the users.

Two factors limited the set of changes applied to the prototypes. The main limiting factor was the time until the next user testing session. The next factor that recommended limiting the set of changes in each prototype was the need to evaluate the impact on the users while maintaining the functionality, as including many changes could make detailed evaluation too tricky.

Due to this prototyping process’s exploratory and discovery nature, it cannot be separated from the testing stage. The experience gained from this project has revealed that the methodology for developing the best prototype cannot be separated into watertight stages. Being user-centered, the process of specification, development, testing, and respecification must be continuous and cannot be disaggregated. Users’ own evaluations generate proposals for modifications, which, in turn, require agility in development, with a very rapid process of specifying new requirements. In many cases, this entire process was reduced to 10 minutes. This extreme speed did not allow for a standard process of requirements gathering, analysis, design, and reimplementation. This section followed a software development paradigm [41] that did not precisely conform to the spiral or rapid prototyping paradigm, although it took many elements from both approaches.

Because of this rapidity, many of the usual thorough error-checking processes cannot be applied. This means that the developed prototypes should not be used for unsupervised external deployments but only for controlled testing. In other words, prototypes should be discarded and only be used to extract fundamental information for constructing the final system.

In short, all these evolutions have made it possible to substantially change the functioning and the layout of the different visual and information input elements.

### Testing

At this point, it is necessary to differentiate between testing and user evaluation. Testing aims to verify the code. Therefore, tests ensure that the code does not contain errors and that the execution provides consistent results. User evaluation seeks validation of the product, that is, the degree of user acceptance of the system. This user evaluation does not seek the correctness of the results, as this is a later phase. Once the product has been developed per the requirements gathered from the users, it can be used by the research team to achieve the project’s objectives.

Focusing on the internal tests, we must distinguish between those carried out before and during the user evaluations. The developments carried out before the evaluations had a short time frame, less than a month, but with the capacity to carry out a sufficient set of tests on the code. These tests detected lexical and syntactic coding faults and even validated results with output verification and acceptable ranges. The testing procedure followed the usual software engineering standards [97]. The code developed before the user evaluations had minor coding errors in specific performances that were difficult to trace. It can be considered good quality code with respect to the specifications previously established for each prototype evolution. However, due to time constraints, code testing was not performed for code modifications made during the evaluations. In this respect, it is essential that the developer involved have extensive experience in programming, in the JavaScript programming language, in Phaser 3, and in the development of web-based systems. The quality of this code varied considerably, although, in general, the code developed could pass the minimum quality requirements.

With regard to user evaluations, focusing on the product development aspect and the acquisition and adjustment of user requirements associated with the system, the procedure had to be more systematic. Follow-up documents were produced and filled in with the subjective evaluation of the user tests. In addition, video recordings were made to allow for a more analytical review of the user tests. In the documents and the video, we did not ask the same questions in all cases, so the users’ answers may have been somewhat biased in some cases. However, we think this bias did not affect the sense of the suggested modifications, since the indications were mostly oriented to help users with the assignment of the different foods to the food group buttons. Only when users got stuck in the procedure for too long was full guidance provided until the process was completed. The evaluation of the reasons for the impossibility of completing the process was analyzed. In some cases, the size of the buttons and the lack of knowledge about the assignment of foods to food groups were problematic.

In general, user tests allowed the prototypes to evolve into the final design, which saved the project a lot of time and money.

Although SMARTCLOTH is conceived as a hardware device rather than a mobile app, its integrated intelligent functionalities—such as real-time conversion of food weights into macronutrient portions, barcode or QR scanning, and automated calorie estimation—are expected to reinforce both glycemic control and dietary adherence. From a clinical perspective, improved precision in dietary monitoring is anticipated to contribute to reductions in HbA_1c_, as demonstrated in studies evaluating technology-based wearable interventions compared with standard care [98]. Beyond glycemic outcomes, SMARTCLOTH could also increase adherence by simplifying complex dietary tasks, reducing the cognitive burden of carbohydrate counting, and making the process more understandable. Usability and clarity of device interfaces have been shown to influence treatment adherence in diabetes care, with user-friendly designs being associated with better long-term engagement [99]. This characteristic is particularly relevant during the early stages after diagnosis, when patients often report errors and insecurity in carbohydrate counting, and an assistive hardware device may function as both an educational and behavioral reinforcement tool. Nevertheless, the translation of these functionalities into measurable clinical outcomes will depend on sustained device engagement, data completeness, and seamless integration into daily routines.

### Methodological Reflections: Limitations and Strengths of the HCD Approach

These reflections on the limitations and strengths of our HCD application provide important considerations for other teams adapting this methodology to their own hardware development projects.

This project presents several strengths that enhance the validity and applicability of its findings. First, the use of an HCD methodology ensured that the prototype was developed based on the real needs and experiences of patients and health care professionals, thereby increasing usability and acceptance. Second, the iterative prototyping and testing process enabled continuous refinements, contributing to a functional and user-friendly system. Third, the inclusion of diverse patient profiles (adolescents with T1DM, working-age adults with T2DM, and older adults with low adherence) provided a comprehensive understanding of different user needs and improved the generalizability of the results.

Nevertheless, several limitations should be acknowledged. The user testing involved a relatively small sample, which may restrict representativeness. Evaluations were conducted in controlled settings rather than during everyday use, limiting inferences about long-term adoption. Finally, the current prototype is oriented toward home use, which may limit its applicability in outdoor contexts where dietary management can be especially challenging.

Although the HCD approach enabled us to gather a wide range of ideas and proposals from patients and professionals, not all of them proved feasible—whether due to technological or economic constraints of the project—or guaranteed a significant clinical impact. This fact is an inherent limitation of participatory processes in complex areas, such as diabetes management, particularly when patients express needs that are difficult to address without a highly active role on their part (eg, motivation). Nevertheless, the iterative nature of the design process and the triangulation with health care professionals allowed us to refine these contributions, prioritizing those that were more feasible and had greater potential applicability. In this way, HCD was useful not only for capturing perceptions and needs but also for guiding a process of selection and refinement oriented toward realistic technological solutions aligned with the precise needs of users.

### Limitations and Adaptability Considerations

The methodology presented reflects our experience with a specific clinical context (diabetes dietary management) and available resources (an academic research team with engineering and clinical expertise). Teams adopting this approach should consider the following:

1. Resource allocation: Our timeline (8 months from mapping to testing) may need adjustment based on team size and funding.
2. Technical expertise: We chose web-based prototyping (Phaser 3) for accessibility; teams with different capabilities might select alternative frameworks.
3. User recruitment: Access to patient engagement boards or clinical partnerships will influence the feasibility of iterative testing.
4. Cultural and linguistic adaptation: Our Spanish-speaking population required specific interface considerations that will differ in other contexts.

The methodology is broadly applicable to other chronic disease management contexts and health care hardware development scenarios. By sharing our experiences, decision-making processes, and lessons learned, we aim to support other multidisciplinary teams in developing user-centered digital health solutions. Future work should explore adaptations of this approach for resource-constrained settings, integration with established clinical workflows, and methods for scaling from prototype to clinical implementation and evaluation.

### Conclusions

This tutorial has provided step-by-step methodological guidance for applying HCD and the Double Diamond model to health care hardware development. Through the SMARTCLOTH case example, we have demonstrated how systematic user engagement, iterative prototyping, and structured usability testing can inform the development of digital health solutions tailored to patient needs. Key methodological contributions of this tutorial include (1) a replicable framework for user needs assessment combining multiple HCD tools (persona, empathy mapping, and customer journey mapping) that can be adapted to different chronic disease contexts; (2) strategies for synthesizing multidisciplinary input from clinical and technical team members through structured cocreation sessions; (3) cost-effective prototyping approaches using web-based frameworks (Phaser3/HTML5) that enable rapid iteration before committing to physical manufacturing; (4) structured usability testing protocols combining quantitative metrics (task completion time and error rates) with qualitative feedback (think-aloud technique) across diverse user populations; and (5) practical guidance on adapting design requirements for users with varying technological literacy levels, including specific accommodations for older adults and users with limited digital experience.

In our specific application to diabetes dietary management, the resulting SMARTCLOTH prototype demonstrated strong usability and user acceptance across 3 distinct user profiles. The iterative development process, guided by continuous patient and professional input, yielded a tool perceived as intuitive and potentially valuable for dietary self-management support.

The methodology presented in this tutorial is broadly applicable to other chronic disease management contexts and health care hardware development scenarios where user-centered solutions are needed. By sharing our experiences, decision-making processes, and lessons learned—including both successes and limitations—we aim to support other multidisciplinary teams in developing effective digital health hardware.

Future applications of this methodology could explore adaptations for different resource settings, integration with existing clinical workflows and electronic health record systems, methods for scaling from prototype to clinical implementation, and approaches for conducting effectiveness evaluations that measure both usability and clinical outcomes. For SMARTCLOTH specifically, next steps include larger-scale usability testing, evaluation of clinical effectiveness on dietary adherence and glycemic control, and development of portable versions suitable for use beyond the home environment.

## Supplementary material

10.2196/75744 Multimedia Appendix 1 General phases in the SMARTCLOTH development project. Adapted from Gasca and Zaragozá, 2021.

10.2196/75744 Multimedia Appendix 2 Different user tests and patients using SMARTCLOTH.

10.2196/75744 Multimedia Appendix 3 Grid of actions for user test 2 task using SMARTCLOTH.

10.2196/75744 Multimedia Appendix 4 Results of In/Out and Strengths, Weaknesses, Opportunities, and Threats.

10.2196/75744 Multimedia Appendix 5 Persona, empathy map, and consumer journey map.

10.2196/75744 Multimedia Appendix 6 Tests 1 and 2 results.

10.2196/75744 Multimedia Appendix 7 Tests 3 and 4 results.

10.2196/75744 Multimedia Appendix 8 Test 5 results.

## Abbreviations

DM: diabetes mellitus
DT: design thinking
HCD: human-centered design
SWOT: Strengths, Weaknesses, Opportunities, and Threats
T1DM: type 1 diabetes mellitus
T2DM: type 2 diabetes mellitus
UNESCO: United Nations Educational, Scientific and Cultural Organization
