# Supplementary figures and images for: SMARTCLOTH Prototype for Dietary Management in Patients With Diabetes Mellitus: Tutorial on Human-Centered Design Methodology for Health Care Hardware Development
Source: J Med Internet Res. 2026 Jan 21;28:e75744. doi: 10.2196/75744 (PMC12826948; doi:10.2196/75744)

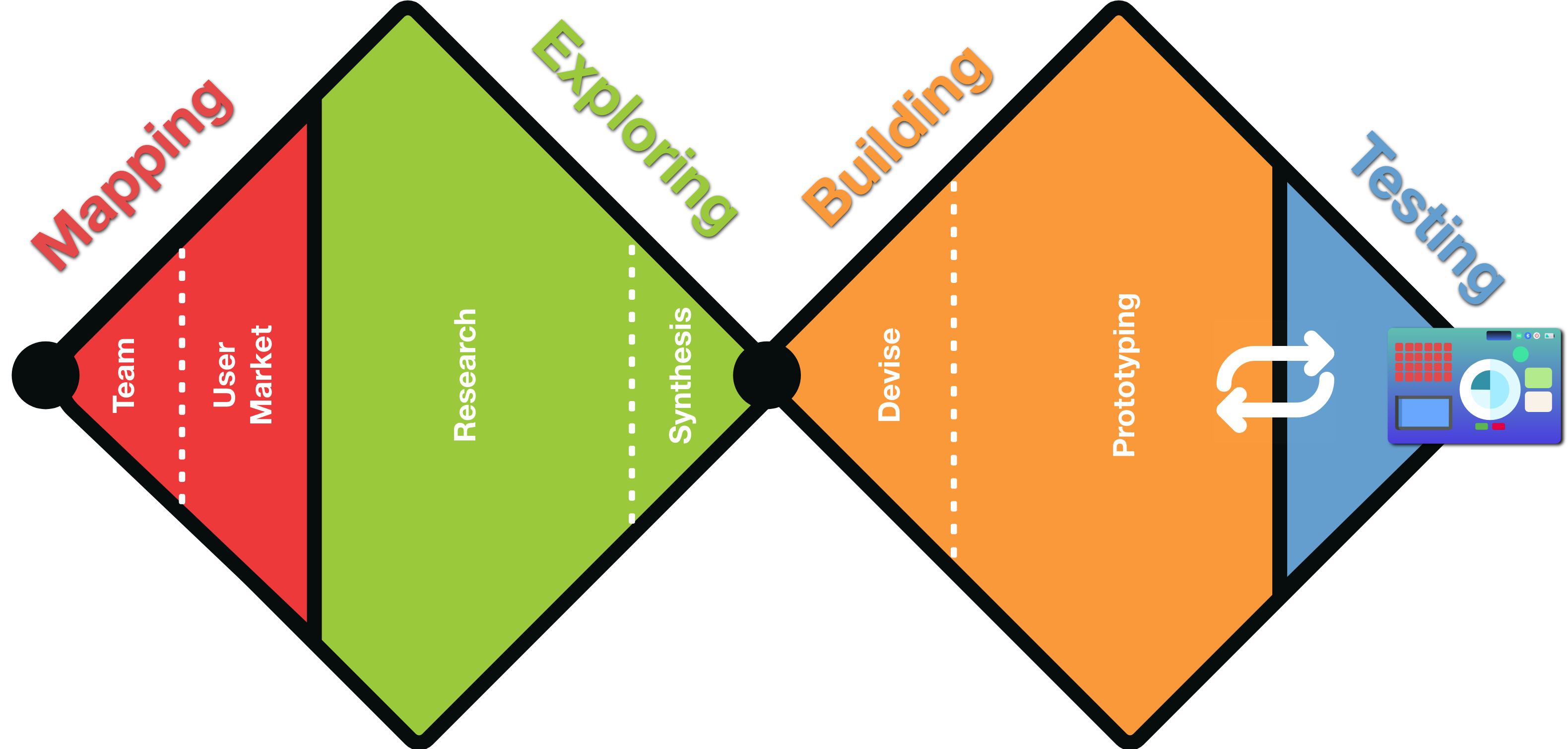

Supplement: Multimedia Appendix 1 [file jmir-v28-e75744-s001.png]

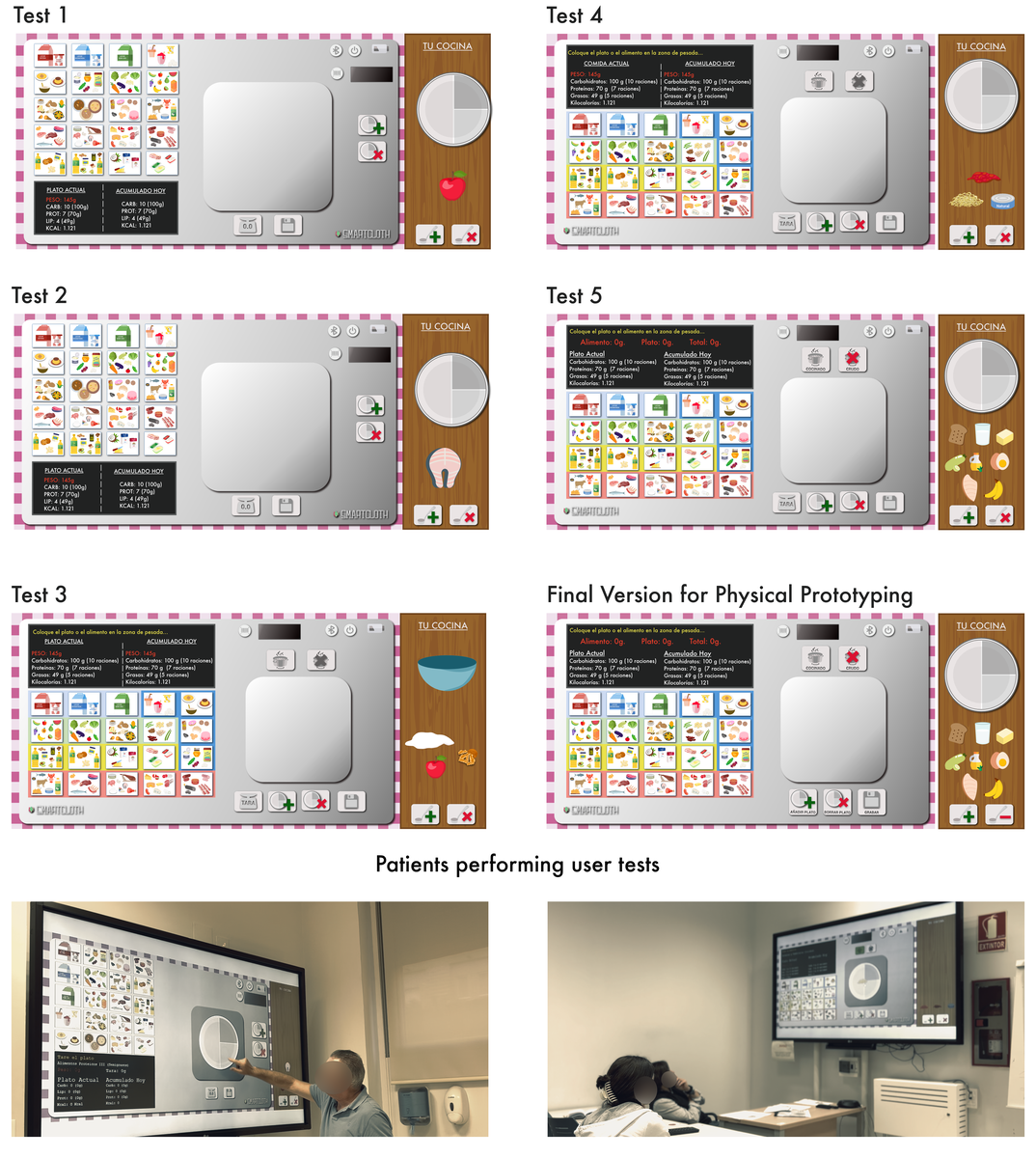

Supplement: Multimedia Appendix 2 [file jmir-v28-e75744-s002.png]

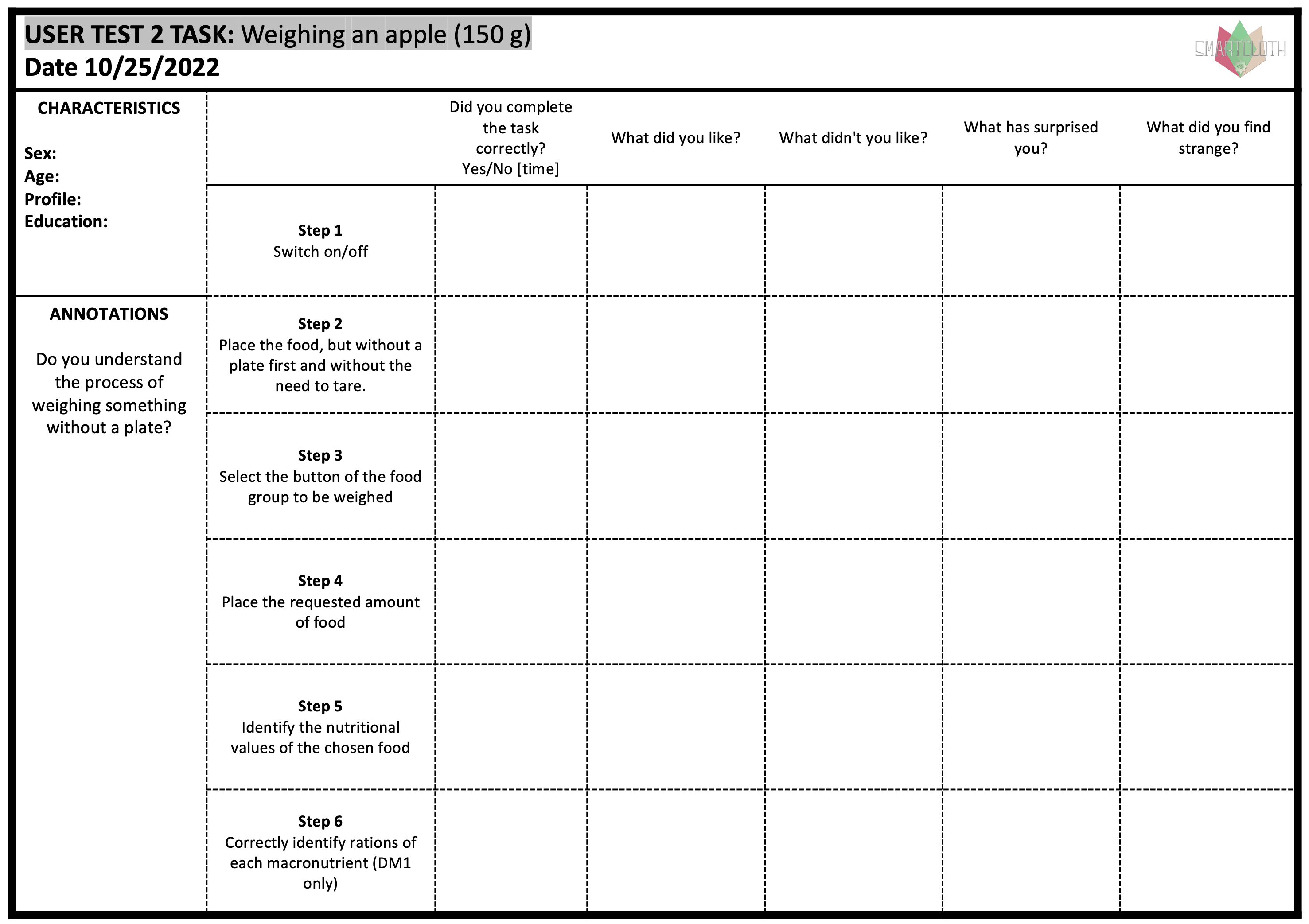

Supplement: Multimedia Appendix 3 [file jmir-v28-e75744-s003.png]

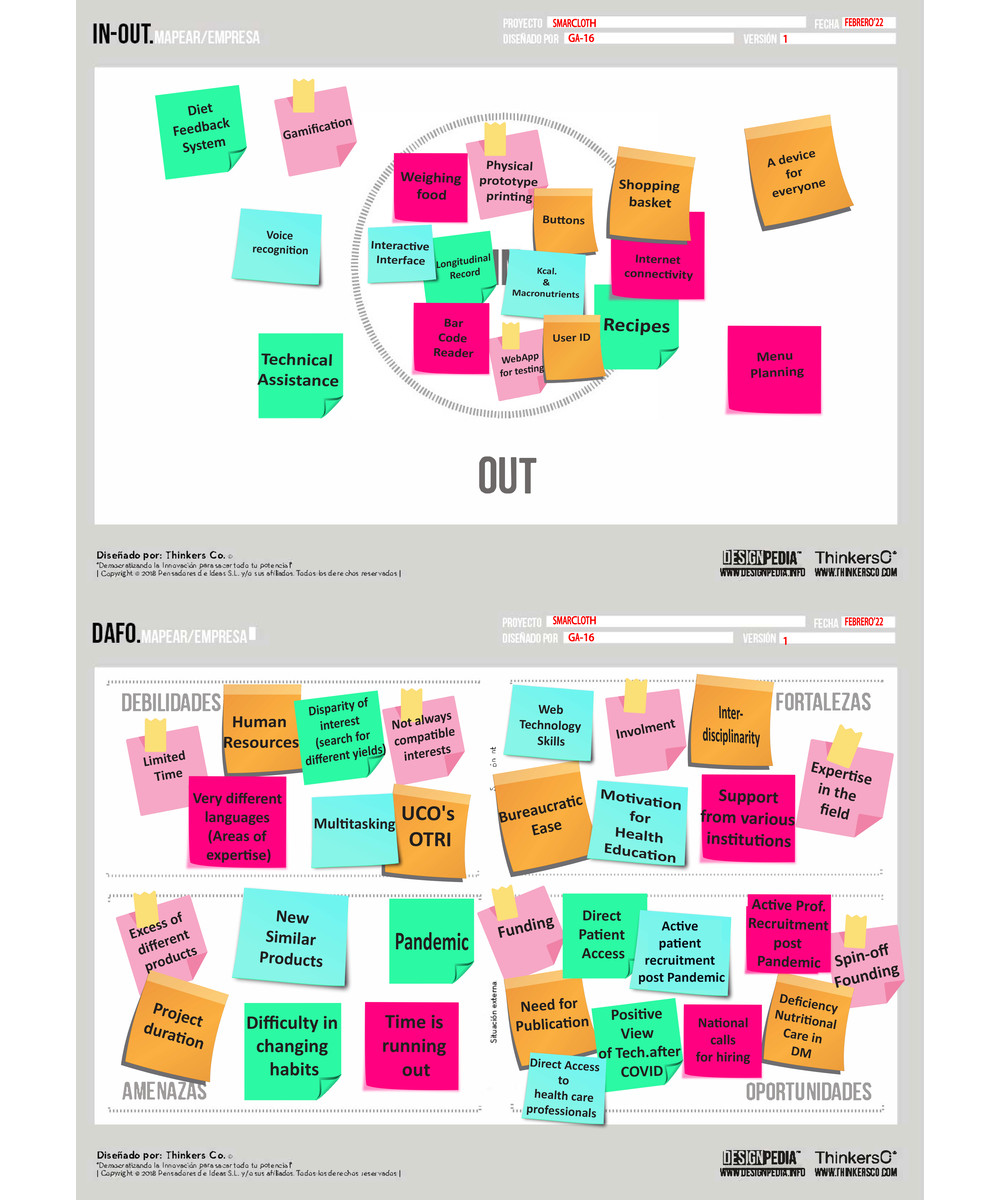

Supplement: Multimedia Appendix 4 [file jmir-v28-e75744-s004.png]
