# Supplementary material for: SMARTCLOTH Prototype for Dietary Management in Patients With Diabetes Mellitus: Tutorial on Human-Centered Design Methodology for Health Care Hardware Development
Source: J Med Internet Res. 2026 Jan 21;28:e75744. doi: 10.2196/75744 (PMC12826948; doi:10.2196/75744)
Supplement: Multimedia Appendix 5 [file jmir-v28-e75744-s005.pdf]

PERSONA. MAPEAR/CLIENTE

PROYECTO SMARTCLOTH FECHA ABRIL'22
DISEÑADO POR GA-16 VERSIÓN Definitiva

CARACTERISTICAS DEMÓGRAFICAS / COMUNIDAD

¿cuál es su edad?¿cómo es su familia?
¿cuál es su trabajo?
¿cuál es su nivel cultural?
¿con quién tiene más confianza?¿quién es la persona en la que se apoya?

- 52 años
- Exceso de peso
- Taxista
- Vive Sola
- Secundarios (algo familiarizada con tecnología)
- Divorciada Hijos independientes

NECESIDADES / MOTIVACIONES

¿cuáles son sus Necesidades / Motivaciones en el Momento / Escenario descrito?

- Problema para las cantidades
- Depende de turnos para comer bien o no
- Mejorar conocimientos alimentación
- No mucho tiempo para planificar o preparar comidas
- No quiere dejar el pan
- Quiere guisos
- Preocupada por cuidarse
- Entender cómo la diabetes afecta a su salud
- Quiere pasarlo bien el día que libra
- Comer mejor fuera de casa
- Manejar el estrés
- Comer mejor fuera de casa
- Mantener vida social y placer por la comida social
- Perder peso

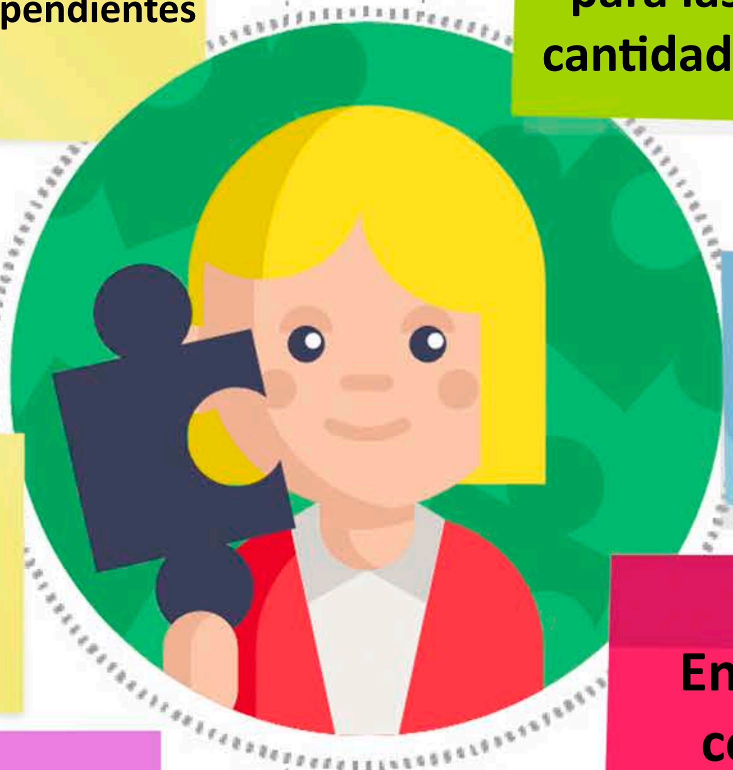

Julia[DM2]

MOMENTO / ESCENARIO

¿cuál es el Momento / Escenario donde le situamos?

- Almuerzo o Cena
- Comida en casa. Con prisas para ir a trabajar
- Almuerzo en el taxi
- Comida familiar con hijos
- Cena en casa. Cansada de todo el día fuera

Diseñado por: Thinkers Co. ©
"Democratizando la Innovación para sacar todo tu potencial"
| Copyright © 2018 Pensadores de Ideas S.L. y/o sus afiliados. Todos los derechos reservados |
DESIGNPEDIA WWW.DESIGNPEDIA.INFO ThinkersCo® WWW.THINKERSCO.COM

MAPA DE EMPATIA. MAPEAR/CLIENTE

PROYECTO: SMARTCLOTH
FECHA: ABRIL'22
DISEÑADO POR: GA-16
VERSIÓN: Definitiva

Julia[DM2]

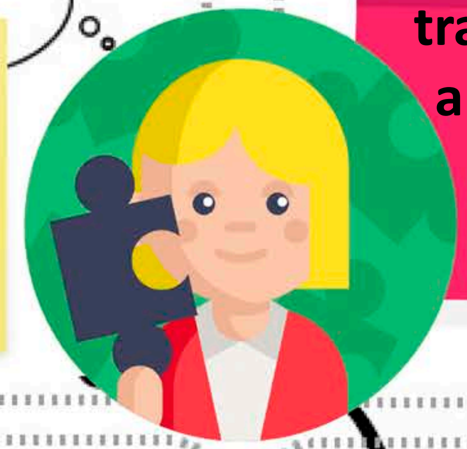

PIENSA

- Es un gran sacrificio
- Bastante tengo en mi vida para preocuparme por otra cosa más
- Hay que evitar el "pinchazo"

DICE

- Todo el día trabajando para ahora pararme a pensar qué cómo
- Hay que estudiarlo y supone tiempo
- Quiero bajar de peso
- Necesito organizar toda esta información (confusa)
- Hay poca comida en el plato

SIENTE

- Miedo (pinchazo) Incertidumbre
- Tiene poco tiempo. Va a todos los sitios corriendo
- Hay poca información (tiempos limitados)
- Motivación inicial disminuye al añadir medicación oral

HACE

- Se informa en Internet y RRSS (recetas)
- Depende del día y de sus turnos
- Se salta la dieta en fiestas y reuniones familiares
- Come mejor o peor
- Busca clínicas privadas

OYE

- A su edad es importante cuidarse
- Debe pensar en su salud para el futuro
- Con la medicación es suficiente
- Información no oficial (vecinas, amigas, etc.)
- No tiene cura
- Mucho sobre medicación y poco sobre alimentación

VE

- La gente no se preocupa tanto por lo que come
- Las personas de su edad alternan mucho
- Se pide mucha comida a domicilio y se cocina poco
- Mucha información confusa
- Conceptos contradictorios

Diseñado por: Thinkers Co. ©
"Democratizando la Innovación para sacar todo tu potencial"
| Copyright © 2018 Pensadores de Ideas S.L. y/o sus afiliados. Todos los derechos reservados |
DESIGNPEDIA WWW.DESIGNPEDIA.INFO ThinkersCo WWW.THINKERSCO.COM

PERSONA. MAPEAR/CLIENTE

PROYECTO SMARTCLOTH
FECHA ABRIL'22
DISEÑADO POR GA-16
VERSIÓN Definitiva

CARACTERISTICAS DEMÓGRAFICAS / COMUNIDAD

¿cuál es su edad? ¿cómo es su familia?
¿cuál es su trabajo?
¿cuál es su nivel cultural?
¿con quién tiene más confianza? ¿quién es la persona en la que se apoya?

- 70 años
- Jubilado
- Primarios (uso escaso tecnología)
- Mujer e hijos
- Problemas de obesidad
- Insulino dependiente

MOMENTO / ESCENARIO

¿cuál es el Momento / Escenario donde le situamos?

- Almuerzo o Cena
- Dicusión con la mujer
- Recorrido largo de la enfermedad

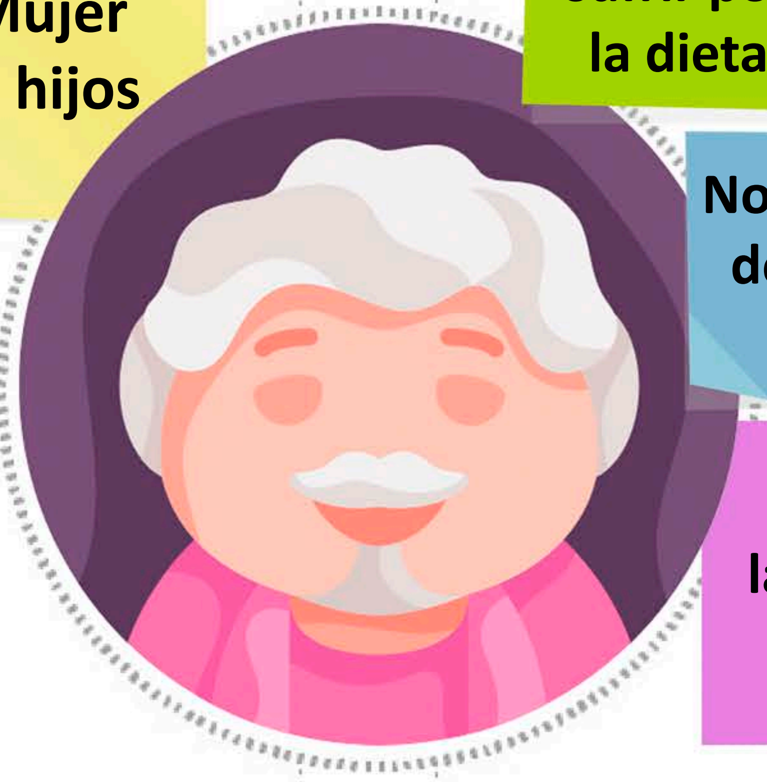

Paco [DM2]

NECESIDADES / MOTIVACIONES

¿cuáles son sus Necesidades / Motivaciones en el Momento / Escenario descrito?

- No quiere sufrir por la dieta
- A veces usa catering
- Inseguridad. Necesita mejorar sus conocimientos (cree que es solo comer lechuga)
- No quiere dejar el pan
- Quiere cocina tradicional
- Mayor control de cantidades y calorías
- Le gusta la comida rápida
- Consume alcohol ocasionalmente
- Solo come un plato (guisos)
- Perder peso
- Le importa la calidad de vida
- Falta de interés
- Vigilar Economía
- Implicar familia en los hábitos
- Le gustan los dulces

Diseñado por: Thinkers Co. ©
"Democratizando la Innovación para sacar todo tu potencial"
| Copyright © 2018 Pensadores de Ideas S.L. y/o sus afiliados. Todos los derechos reservados |

DESIGNPEDIA™ WWW.DESIGNPEDIA.INFO
ThinkersCo® WWW.THINKERSCO.COM

MAPA DE EMPATIA. MAPEAR/CLIENTE

PROYECTO SMARTCLOTH
FECHA ABRIL'22
DISEÑADO POR GA-16
VERSIÓN Definitiva

Paco [DM2]

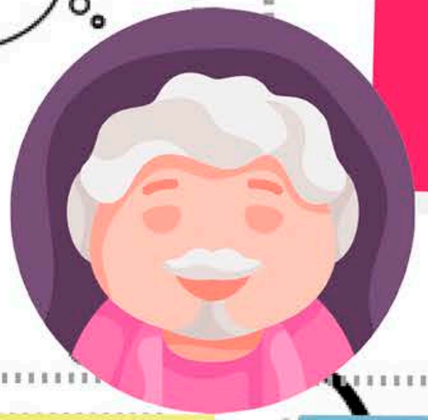

PIENSA

- No me entero bien
- A estas alturas, no voy a cambiar mi forma de comer
- ¿Ejercicio a mis años?
- Es un gran sacrificio
- Método complejo y duro (estricto)
- De algo hay que morir

DICE

- Llevo toda mi vida comiendo así
- No me entero bien. Es muy difícil
- Que se entere bien mi mujer
- Yo me siento como siempre
- Precaución por hipoglucemia

SIENTE

- Está muy cansado. No quiere complicaciones
- Le gusta comer (comer sano aburre)
- No quiere renunciar a lo poco que le queda de disfrutar
- Hipoglucemias nocturnas
- Cambiaré si empeoro
- Desconfianza Incertidumbre

HACE

- Delega responsabildad en la esposa
- En general, no ha cambiado su estilo de vida y no sigue la dieta
- Discute con su mujer por las comidas
- Sigue su vida como si nada hubiera pasado

OYE

- A su edad es importante cuidarse
- Todo el mundo está muy ocupado
- Conceptos ajenos y complicados
- Mucho sobre medicación y poco sobre alimentación

VE

- Otros familiares y amigos con DM2 siguen con su vida igual
- A veces, su mujer y él están muy solos
- Otros amigos salen y alternan sin pensar en dietas

Diseñado por: Thinkers Co. ©
"Democratizando la Innovación para sacar todo tu potencial"
| Copyright © 2018 Pensadores de Ideas S.L. y/o sus afiliados. Todos los derechos reservados |

DESIGNPEDIA™ WWW.DESIGNPEDIA.INFO
ThinkersCo® WWW.THINKERSCO.COM

PERSONA. MAPEAR/CLIENTE

PROYECTO SMARTCLOTH
FECHA ABRIL'22
DISEÑADO POR GA-16
VERSIÓN Definitiva

CARACTERISTICAS DEMÓGRAFICAS / COMUNIDAD

¿cuál es su edad?¿cómo es su familia?
¿cuál es su trabajo?
¿cuál es su nivel cultural?
¿con quién tiene más confianza?¿quién es la persona en la que se apoya?

- 17 años
- Estudiante
- Secundaria (buena relación tecnología)
- Necesita Padres para comida Come fuera de casa
- Buen manejo insulina

NECESIDADES / MOTIVACIONES

¿cuáles son sus Necesidades / Motivaciones en el Momento / Escenario descrito?

- Vida Social
- Poder regular con resto de comidas
- Lo entiende bien
- Cena mal
- Conocer el impacto en su salud del alcohol
- Le gusta la comida rápida
- Disfrutar y divertirse mientras come

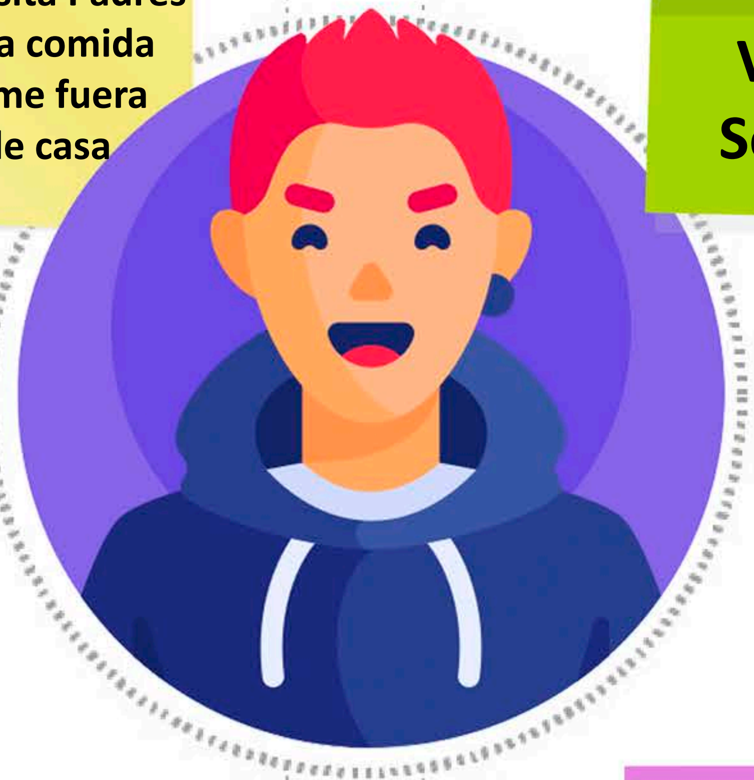

Kevin [DM1]

MOMENTO / ESCENARIO

¿cuál es el Momento / Escenario donde le situamos?

- Cena en casa
- Consume alcohol los fines de semana
- Ha comido fuera sin seguir la dieta
- Conflictos familiares puntuales

Diseñado por: Thinkers Co. ©
"Democratizando la Innovación para sacar todo tu potencial"
| Copyright © 2018 Pensadores de Ideas S.L. y/o sus afiliados. Todos los derechos reservados |
DESIGNPEDIA™ WWW.DESIGNPEDIA.INFO
ThinkersCo® WWW.THINKERSCO.COM

MAPA DE EMPATIA. MAPEAR/CLIENTE

PROYECTO SMARTCLOTH
FECHA ABRIL'22
DISEÑADO POR GA-16
VERSIÓN Definitiva

Kevin [DM1]

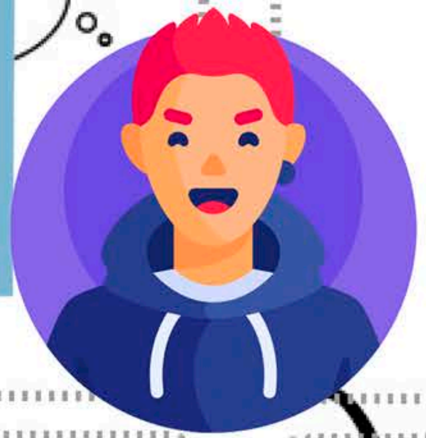

PIENSA

Dieta es un rollo

Quiero disfrutar con mis amigos y no ser el rarito

Si tengo una bajada, puedo compensar comiendo lo que me gusta

Controlar siempre la glucemia es muy complejo

DICE

Quiero salir con mis amigos y pasarlo bien

No tengo el control de mi enfermedad

SIENTE

Es JOVEN. Quiere divertirse

Por mucho que haga, siempre estoy descontrolado

¿Por qué no puedo comer como los demás?

HACE

Si tengo una hiopoglucemia, come lo que quiere

Sale con sus amigos y va a sitios de comida rápida

OYE

La gente de su edad sale, se lo pasan bien y comen donde quieren

¿Eso te vas a comer?

Si te pasas, te pones más insulina y ya está

VE

Si como diferente, me miran raro

Sus amigos salen y no se preocupan por lo que comen

Diseñado por: Thinkers Co. ©
"Democratizando la Innovación para sacar todo tu potencial"
| Copyright © 2018 Pensadores de Ideas S.L. y/o sus afiliados. Todos los derechos reservados |

DESIGNPEDIA WWW.DESIGNPEDIA.INFO ThinkersCo WWW.THINKERSCO.COM

CUSTOMER JOURNEY MAP. MAPEAR/CLIENTE

PROYECTO SMARTCLOTH — FECHA ABRIL'22

DISEÑADO POR GA-16 — VERSIÓN DEFINITIVA

ANTES — DURANTE — DESPUÉS

ACCIONES DEL USUARIO	1) Hecer Menú semanal o mensual	2) Hacer la Compra	3) Decidir qué Comer Hoy	4) Cocinar lo que quiero Comer	5) Servir las raciones	6) Ingerir las raciones	7) Recoger	8) Limpiar Restos	9) Pensar la siguiente comida	10) Revisión en consulta
Puntos de contacto	Personal sanitario. Educación	Supermercado Anuncios TV Publicidad	Cocina. Despensa	Cocina	Cocina o Comedor	Cocina o Comedor	Cocina o Comedor	Cocina	Casa	Consulta sanitario
¿Qué está pasando? ¿Qué está sintiendo?	Debe seguir dieta por raciones o método del plato<br>No entiende bien los sistemas	Va al súper a hacer la compra<br>Debe elegir entre productos<br>Puede ser útil si hay formación<br>Lucha interna	Depende de si hay menú o no<br>Elegir con dieta y dudas siempre es doloroso<br>Incompatible con comidas tradicionales	Puede cocinar el usuario<br>O delegar en familiar o en cuidador<br>Problema con cantidad de aceite	Difícil ajustar las cantidades<br>Problemas con el pesaje<br>Difícil controlar pan y fruta<br>Muy difícil con guisos	Ingerir alimentos no adecuados<br>¿Temor? ¿Culpa? ¿Placer?<br>¿Indiferencia?	Pueden hacerlo o delegarlo en familiares o cuidadores<br>La percepción varía en función de quien realice la actividad, pero en general, es bien aceptada		Una dificultad independiente de quien la realice	Siempre es doloroso<br>No pérdida o ganancia de peso<br>Incremento HbAc1

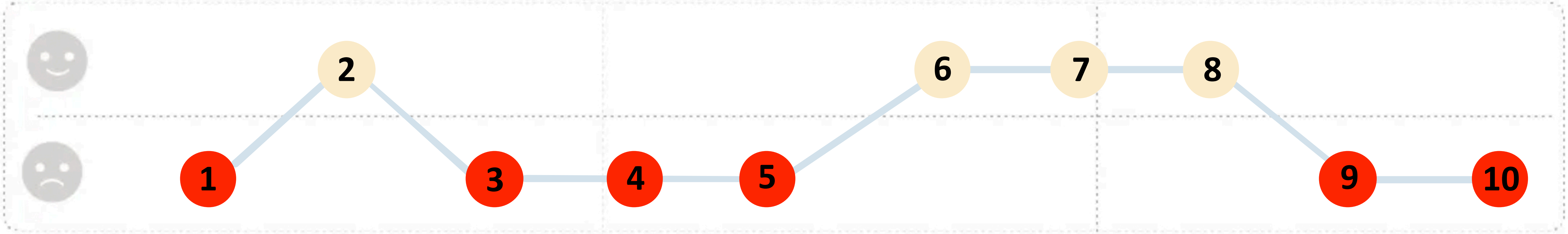

Diseñado por: Thinkers Co. ©
"Democratizando la Innovación para sacar todo tu potencial"
| Copyright © 2018 Pensadores de Ideas S.L. y/o sus afiliados. Todos los derechos reservados |

DESIGNPEDIA™ WWW.DESIGNPEDIA.INFO
ThinkersCo® WWW.THINKERSCO.COM

CUSTOMER JOURNEY MAP. MAPEAR/CLIENTE

PROYECTO SMARTCLOTH
FECHA ABRIL'22
DISEÑADO POR GA-16
VERSIÓN DEFINITIVA

	ANTES	DURANTE		DESPUÉS	
ACCIONES DEL USUARIO	1) Primera Consulta	2) Adaptación	3) Información métodos	4) Cambios en la dieta	5) Seguimiento
Puntos de contacto	Presencial en consulta	Presencial o telefónica	Presencial en consulta	Telefónica	Presencial o telefónica
¿Qué está pasando? ¿Qué está sintiendo?	Muchas dudas y miedos<br>Desconfianza<br>Soledad	Conceptos se van aclarando<br>Más calma<br>Autoconfianza<br>Autoeficacia<br>Motivación<br>Ejercicio físico	Información con métodos visuales<br>Diabetes a la carta [Plato Harvard]<br>Interés	Dudas concretas<br>Seguridad	Dudas concretas<br>Confianza<br>Normalización<br>Busca nuevas herramientas<br>Nutricionista privado

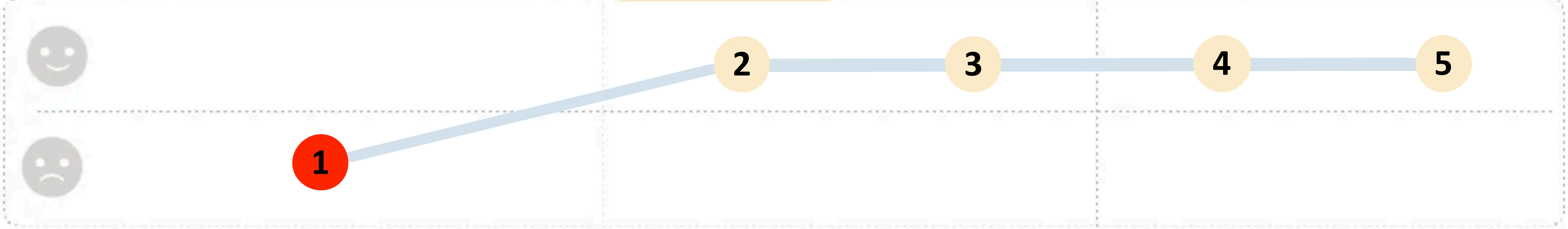

Diseñado por: Thinkers Co. ©
"Democratizando la Innovación para sacar todo tu potencial"
| Copyright © 2018 Pensadores de Ideas S.L. y/o sus afiliados. Todos los derechos reservados |

DESIGNPEDIA WWW.DESIGNPEDIA.INFO
ThinkersCo WWW.THINKERSCO.COM

CUSTOMER JOURNEY MAP. MAPEAR/CLIENTE

PROYECTO: SMARTCLOTH
FECHA: ABRIL'22
DISEÑADO POR: GA-16
VERSIÓN: DEFINITIVA

	ANTES	DURANTE	DESPUÉS
ACCIONES DEL USUARIO	1) Primera Consulta	2) Consulta de Control	3) Tras indicación de tratamiento con insulina
Puntos de contacto	Presencial<br>30 minutos	Presencial<br>10-15 minutos	Presencial<br>10-15 minutos
¿Qué está pasando?<br>¿Qué está sintiendo?	Se explica el método del plato<br><br>Motivación o incertidumbre y rechazo	No ha mejorado o alcanzado metas fijadas<br><br>Debe contar kilocalorías<br><br>Rechazo al pesado de alimentos<br><br>Recomendaciones muy generales	Explican el método por raciones<br><br>Explican manejo glucómetro e insulina<br><br>Agobio<br><br>Sobrepasados por la cantidad de información

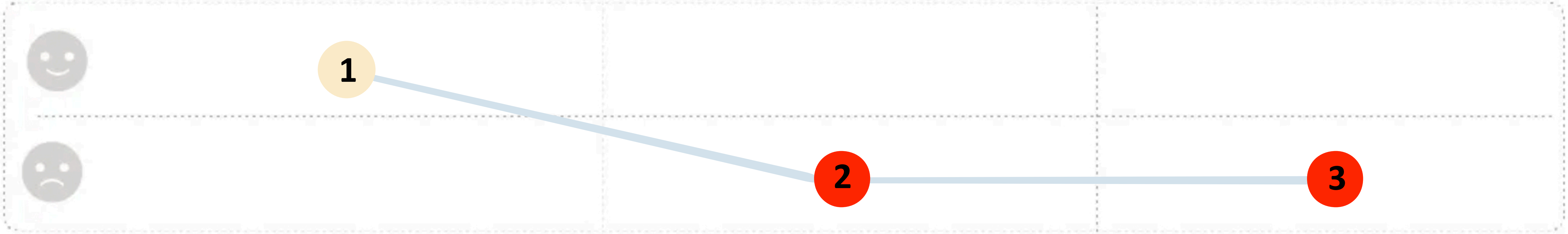

Diseñado por: Thinkers Co. ©
"Democratizando la Innovación para sacar todo tu potencial"
| Copyright © 2018 Pensadores de Ideas S.L. y/o sus afiliados. Todos los derechos reservados |

DESIGNPEDIA™ WWW.DESIGNPEDIA.INFO
ThinkersCo® WWW.THINKERSCO.COM
