# Supplementary material for: SMARTCLOTH Prototype for Dietary Management in Patients With Diabetes Mellitus: Tutorial on Human-Centered Design Methodology for Health Care Hardware Development
Source: J Med Internet Res. 2026 Jan 21;28:e75744. doi: 10.2196/75744 (PMC12826948; doi:10.2196/75744)
Supplement: Multimedia Appendix 6 [file jmir-v28-e75744-s006.docx]

| Supplementary Table 1. Test 1 & 2 results | | | | | | | | | |
| --- | --- | --- | --- | --- | --- | --- | --- | --- | --- |
| User | **Test** | **Tries** | **Average Attempts Time** | **Last Try** | **Explanations needed** | **Errors** | **Positive reinforcements needed** | **Direct helps**  **needed** | **Indirect helps**  **needed** |
| P1.1 | 1 | 2 | 43’’ | 56’’ | 2 | 0 | 1 | 0 | 1 |
|  | 2 | 1 | 1’43’’ | 1’43’’ | 1 | 0 | 5 | 1 | 1 |
| P1.2 | 1 | 2 | 52’’ | 56’’ | 2 | 0 | 3 | 0 | 2 |
|  | 2 | 1 | 1’07’’ | 1’07’’ | 1 | 0 | 2 | 0 | 1 |
| P1.3 | 1 | 2 | 51’’ | 50’’ | 2 | 0 | 1 | 1 | 2 |
|  | 2 | 1 | 1’37’’ | 1’37’’ | 1 | 0 | 3 | 1 | 1 |
| P1.4 | 1 | 1 | 2’31’’ | 2’31’’ | 1 | 0 | 3 | 0 | 2 |
|  | 2 | 2 | 1’30’’ | 2’04’’ | 2 | 0 | 2 | 1 | 3 |
| P1.5 | 1 | 1 | 1’38’’ | 1’38’’ | 1 | 0 | 3 | 0 | 3 |
|  | 2 | 2 | 1’23’’ | 51’’ | 2 | 0 | 5 | 2 | 1 |
| P2.1 | 1 | 2 | 1’20’’ | 53’’ | 2 | 0 | 1 | 2 | 2 |
|  | 2 | 1 | 1’19’’ | 1’19’’ | 1 | 0 | 5 | 0 | 0 |
| P2.2 | 1 | 1 | 1’49’’ | 1’49’’ | 1 | 0 | 3 | 1 | 2 |
|  | 2 | 1 | 1’08’’ | 1’34’’ | 1 | 0 | 4 | 2 | 1 |
| P3.1 | 1 | 3 | 2’31’’ | 1’32’’ | 3 | 1 | 1 | 4 | 2 |
|  | 2 | 2 | 2’30’’ | 2’30’’ | 2 | 0 | 4 | 4 | 4 |
|  | 2 | 2 | 1’44’’ | 1’44’’ | 2 | 0 | 3 | 1 | 1 |
