# Supplementary material for: SMARTCLOTH Prototype for Dietary Management in Patients With Diabetes Mellitus: Tutorial on Human-Centered Design Methodology for Health Care Hardware Development
Source: J Med Internet Res. 2026 Jan 21;28:e75744. doi: 10.2196/75744 (PMC12826948; doi:10.2196/75744)
Supplement: Multimedia Appendix 7 [file jmir-v28-e75744-s007.docx]

| Supplementary Table 2. Test 3 & 4 results | | | | | | | | | |
| --- | --- | --- | --- | --- | --- | --- | --- | --- | --- |
| User | **Test** | **Tries** | **Average Attempts Time** | **Last Try** | **Explanations needed** | **Errors** | **Positive reinforcements needed** | **Direct helps**  **needed** | **Indirect helps**  **needed** |
| P1.1 | 3 | 1 | 4’04’’ | 4’04’’ | 1 | 0 | 4 | 5 | 2 |
|  | 4 | 1 | 3’39’’ | 3’39’’ | 1 | 0 | 4 | 2 | 5 |
| P1.2 | 3 | 1 | 2’57’’ | 2’57’’ | 1 | 0 | 3 | 2 | 2 |
|  | 4 | 1 | 3’11’’ | 3’11’’ | 1 | 0 | 2 | 2 | 3 |
| P1.3 | 3 | 1 | 3’10’’ | 3’10’’ | 1 | 0 | 5 | 2 | 2 |
|  | 4 | 1 | 3’00’’ | 3’00’’ | 1 | 0 | 3 | 3 | 4 |
| P1.4 | 3 | 2 | 1’29’’ | 1’27’’ | 2 | 0 | 4 | 1 | 0 |
|  | 4 | 3 | 1’27’’ | 1’28’’ | 2 | 1 | 2 | 2 | 3 |
| P1.5 | 3 | 2 | 2’26’’ | 1’48’’ | 2 | 1 | 5 | 1 | 3 |
|  | 4 | 1 | 4’48’’ | 4’48’’ | 1 | 0 | 6 | 3 | 1 |
| P2.1 | 3 | 1 | 3’00’’ | 3’00’’ | 1 | 0 | 4 | 2 | 0 |
|  | 4 | 1 | 5’31’’ | 5’31’’ | 1 | 0 | 4 | 1 | 2 |
| P2.2 | 3 | 2 | 2’11’’ | 1’52’’ | 2 | 1 | 4 | 5 | 0 |
|  | 4 | 1 | 2’57’’ | 2’57’’ | 1 | 0 | 5 | 2 | 5 |
| P3.1 | 3 | 2 | 2’38’’ | 2’19’’ | 2 | 0 | 9 | 6 | 4 |
|  | 4 | 1 | 3’17’’ | 3’17’’ | 1 | 0 | 6 | 2 | 4 |
