# Supplementary material for: SMARTCLOTH Prototype for Dietary Management in Patients With Diabetes Mellitus: Tutorial on Human-Centered Design Methodology for Health Care Hardware Development
Source: J Med Internet Res. 2026 Jan 21;28:e75744. doi: 10.2196/75744 (PMC12826948; doi:10.2196/75744)
Supplement: Multimedia Appendix 8 [file jmir-v28-e75744-s008.docx]

| Supplementary Table 3. Test 5 results | | | | | | | | | |
| --- | --- | --- | --- | --- | --- | --- | --- | --- | --- |
| User | **Test** | **Tries** | **Average Attempts Time** | **Last Try** | **Explanations needed** | **Errors** | **Positive reinforcements needed** | **Direct helps**  **needed** | **Indirect helps**  **needed** |
| P1.1 | 5 | 3 | 2’10’’ | 4’18’’ | 2 | 0 | 6 | 1 | 3 |
| P1.2 | 5 | 2 | 5’38’’ | 9’13’’ | 1 | 0 | 4 | 2 | 7 |
| P1.3 | 5 | 1 | 13’10’’ | 13’10’’ | 1 | 1 | 6 | 2 | 3 |
| P1.4 | 5 | 1 | 9’50’’ | 9’50’’ | 1 | 0 | 3 | 0 | 5 |
| P1.5 | 5 | 1 | 10’21’’ | 10’21’’ | 1 | 1 | 6 | 4 | 3 |
| P2.1 | 5 | 1 | 10’10’’ | 10’10’’ | 1 | 0 | 6 | 5 | 5 |
| P2.2 | 5 | 2 | 7’31’’ | 12’42’’ | 2 | 1 | 8 | 7 | 8 |
| P3.1 | 5 | 2 | 8’34’’ | 12’38’’ | 2 | 1 | 9 | 9 | 11 |
